# Forest carbon removal potential and sustainable development in Japan

**DOI:** 10.1038/s41598-024-51308-z

**Published:** 2024-01-05

**Authors:** Bingqi Zhang, Janaki Imbulana Arachchi, Shunsuke Managi

**Affiliations:** Urban Institute and School of Engineering, Kyushu University, 744 Motooka, Nishi-ku, Fukuoka, 819-0395 Japan

**Keywords:** Sustainability, Climate-change mitigation

## Abstract

Forests play a crucial role in mitigating climate change and reducing emissions as a major carbon sink. However, its value in removing carbon dioxide (CO_2_) from the atmosphere is always underestimated in natural capital (NC) accounting and sustainability assessments. This study predicted Japan’s forest CO_2_ removal by afforestation and forest management and its monetary value until 2042 from national to gridded level, with statistical data and complementary satellite data products, and explored how that CO_2_ removal will contribute to sustainable development under the inclusive wealth (IW) framework. The results show that: (1) the annual CO_2_ removal by forests has the potential to offset 15.3% of the emission and increase NC by 6.8% in Japan, significantly contributing to carbon neutrality and IW growth; (2) the total CO_2_ removal in exiting forests will peak at around 2030 and then decrease, but expanding afforestation could offset that decrease in later years; (3) the spatial distribution patterns of IW and forest CO_2_ removal are opposite. This indicates a national carbon trading market could create new wealth for rural communities where vast forests exist, and then effectively balance the inequal urban–rural development in Japan. The explicit spatial information of this study could provide valuable information for differentiating policy priorities of forestry planning and sustainable development in different local communities.

## Introduction

The removal of vast amounts of carbon dioxide from the atmosphere, as well as drastic cuts in emissions, are essential to avoid dangerous climate change. Both IPCC reports^1^ and the Paris Agreement^2^ recognize that climate change mitigation goals cannot be achieved without a substantial contribution from forests. As the forest climate mitigation strategies: afforestation, reforestation, improved forest management, and avoided forest conversion play a critical role in reducing greenhouse gas (GHG) emissions^1,3^. Thus, monitoring and predicting the potential of CO_2_ removal in both managed and new-planted forests is increasingly important for climate policy and the various actors developing nature-based solutions^4^.

As a globally significant GHG emitter, Japan also pledged to achieve net-zero emissions by 2050 which sets Japan on a course to become Carbon Neutral in 30 years^5^. In terms of that the Japanese government issued the “Japan Greenhouse Gas Emission Reduction/Removal Certification Scheme” (J-credit scheme)^6^. Under this scheme, the government certifies the amount of GHG (mainly CO_2_) reduced by introducing renewable energy and energy-saving equipment or removed through the forest sink as “credit”. The credits could be traded voluntarily between J-credit creators and buyers, which forms an emission trading market within Japan. The construction of a carbon trading system will also encourage emission reduction and removal activities by increasing the emission cost^7^. Notably, J-credit created from forests includes CO_2_ removal by implementing two forest activities: afforestation (AR) and forest management (FM). Meanwhile, aging and depopulation in rural areas and wealth accumulation leaning towards metropolitan areas enhance the regional inequality within Japan and lead to unsustainable problems in rural communities^8–10^. Since rural and mountainous areas are rich in forests, accounting for 60% of the national total forest area, the trading of J-credits generated from forest CO_2_ removal could increase wealth and be linked with sustainability there.

Concerning sustainability issues, the basic debate is about the substitutability of different forms of capital and the notions of strong versus weak sustainability. Strong sustainability requires that all major classes of assets are non-declining through time whereas weak sustainability only requires that human well-being is not declining over time. In principle, it is possible to satisfy weak sustainability with declining natural capital (NC) as long as other forms of capital can substitute for natural capital to maintain well-being^11^. Inclusive wealth (IW) is firmly aligned with weak sustainability, as one of the alternatives to the gross domestic product (GDP) in response to the Beyond GDP movement in “Our Common Agenda”^12^. Compared to other alternatives, IW offers a better approach to assess sustainability, since it provides a comprehensive measurement of stock assets, that are related to the potential intergenerational well-being^13,14^. In a practical setting, it estimates the monetary value of human, produced, and natural capital^15^. Especially, the IW framework acknowledges the indispensable role of natural capital in promoting sustainability, since it provides not only direct and indirect provision of well-being but also represents an essential pool of resources that can induce the growth of other capital assets. In recent years, the IW framework has been widely used for the trajectory assessment and long-term projection of sustainability for countries as well as local municipalities^16–19^, implying that the IW framework provides an effective tool to assess how a community performs in terms of sustainable development.

Though there is no current theoretical consensus on the definition of a sustainable community^20^, according to the Egan report for sustainable communities^21^, sustainable communities are defined as communities that meet the different needs of present and future residents and other users and contribute to a high-quality life. They achieve this by making effective use of natural resources, enhancing the environment, promoting social networks and inclusion, and strengthening economic prosperity. It also emphasizes that natural resources are a crucial component in making sustainable communities. Many scholars explored how social factors contribute to the development of sustainable communities by promoting environment-friendly behavior. For example, a meta-analysis indicates a global emissions reduction potential of 0.35 Gt-CO_2_ yr^−1^ through interventions aimed at behavioral change in residential energy use^22^; higher educational levels are associated with an increase in sustainable energy consumption^23^; environmentally friendly behaviors and sustainable communities are also determined by high-quality social capital (i.e., higher social trust and greater interaction with neighbors and communities)^24–26^. Meanwhile, industries will also be influenced to environment-friendly solutions to reduce GHG emissions if the carbon price becomes high enough rather than paying allowances^27^. While those studies focus more on “energy communities”, exploring the efforts to reduce emissions, but tend to ignore activities to offset them. In practice, it has been pointed out that the activities of forest enterprises on sustainable forest management deserve to be noticed and the value addition of forests are significant to community development^28^. And a trading market will also encourage CO_2_ removal activities^6^ as we mentioned before. These kinds of transitioning to sustainable and environment-friendly industrialization would imply a positive impact on cities and human settlements where they are located, thus contributing to UN Sustainable Development Goals 11 (SDGs)^29^.

The Japanese government highlighted the importance of forest products including carbon removal credits to revitalize rural communities in hilly and mountainous area^30^, where IW is decreasing indicating an unsustainable development^9^. Meanwhile, the IW framework can portray the whole capacity of a country/community’s assets that serve both direct and indirect purposes of human well-being covering both market and nonmarket goods and services, including natural resources and environmental services^31^. However, the forest is a major resource of renewable natural capital and has historically been underpriced. Especially, the value of CO_2_ removal and climate mitigation of forest capital is being ignored in the Inclusive Wealth Report (IWR)^16^ and related studies. Therefore, it is prior and crucial to predict Japan’s forest CO_2_ removal potential at a fine spatial scale and to value it in monetary terms by putting it in the context of both the J-credit trading market and the IW framework, for a better understanding on how forest sink will contribute to sustainable development of local communities. The disaggregated estimation could further help to better understand how the forest sink will contribute to local communities’ sustainable development.

Although many previous studies have measured forest carbon sequestration (FCS) globally, some are limited to particular forest type^32,33^, some studies focus on historical patterns rather than the future potential^34^, and some lack disaggregated measurement^35^. Furthermore, literature on the monetary value of FCS is also limited at the country level except for some Western countries and China^36^. Despite Japan having the largest proportion of land area under forests among high-income countries in the world, surprisingly there are very few studies that have assessed the monetary value of Japan’s forest CO_2_ removal^37^. Existing literature for Japan either focuses on the historical tracking of forest carbon stock and flux^38–41^, or the projection of the future sequestration for the case study^42^ and by taking the country as a whole^43^. In addition, none of them has involved the related value of afforestation. Accordingly, existing studies are insufficient to identify the potential monetary value of Japan’s CO_2_ removal by (1) considering both AR and FM, (2) downscaling the measurement to the municipal level, and (3) integrating the forest CO_2_ valuation with sustainable indicators.

To fill this gap, this study objects to contribute to the literature on Japan’s forestry and its potential to remove carbon emissions in three ways: (1) to provide a comprehensive analysis of the CO_2_ removal potential of Japan’s forest management and afforestation activities, (2) to estimate the CO_2_ removal potential from forests at a spatial-explicit level (pixel- and municipal levels), and (3) to examine the contribution of the forest CO_2_ removal to sustainable communities in the country. We first reconstructed the forest volume growth and carbon sequestration model with updated regional-specific parameters under the J-credit scheme based on the IPCC Guidelines, and then projected the CO_2_ removal until 2042 from FM and AR for each prefecture of Japan with the national forest inventory (NFI) data. Secondly, we downscaled the estimation to 100 m—grid level by integrating remote sensing (RS) products, including land cover and biomass data. Thus, we analyzed the temporal trend and spatial heterogeneity of the forest CO_2_ removal potential across the whole country. To the best of our knowledge, this is the first study examining the potential of future forest CO_2_ removal in Japan at four levels: country, prefecture, municipality, and pixel. Lastly, we elaborated on how the potential of forest CO_2_ removal will affect sustainable communities by integrating IW measurements with it.

## Results and discussion

### The CO_2_ removal potential through FM and AR in the future

**Figure 1:**
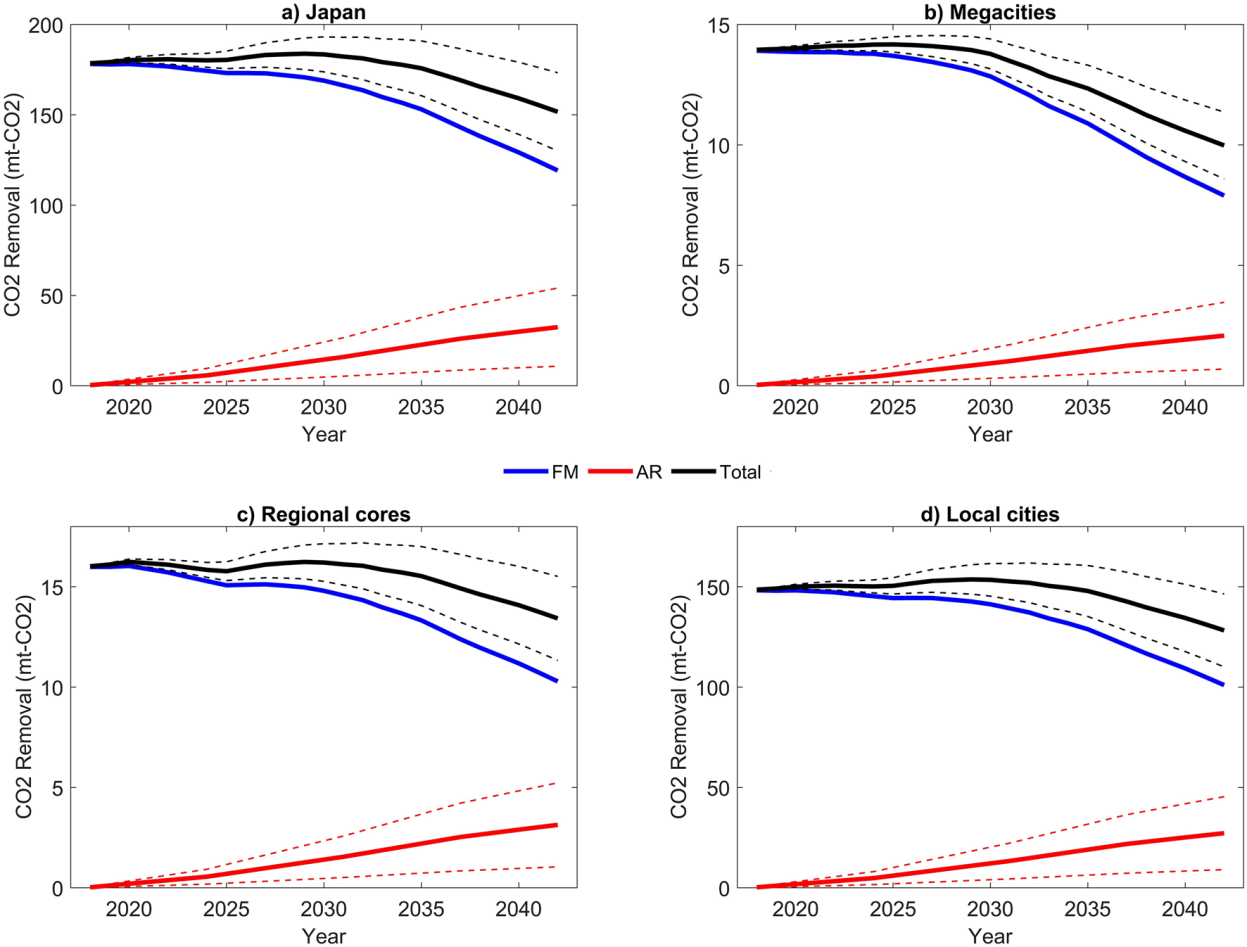
The projection of CO_2_ removal by forests in (**a**) Japan, (**b**) megacities, (**c**) regional cores, and (**d**) local cities. The figure was generated with MATLAB 2023a (https://ch.mathworks.com/products/matlab.html). Dashed lines indicate the results with the upper and lower values of removal by AR, by assuming different levels of afforestation area.

Figure 1a illustrates the nationwide trend of the potential of annual CO_2_ removal from 2018 to 2042 in Japan. The trends for three regions of different levels of urban concentration are also shown in Fig. 1. Specifically, megacities refer to the metropolitan areas centered by Tokyo, Osaka, and Nagoya; regional cores include areas around other important cities of Sapporo, Sendai, Hiroshima, and Fukuoka; other cities and prefectures are classified as local cities (see the map of regional categories supplementary Fig. S1).

Table 1 also shows the 25-year annual average CO_2_ removal value for Japan and these regions. According to the National Greenhouse Gas Inventory Report of Japan, the total GHG emission of Japan in 2020 was 1150 Mt-CO_2_ in 2020^41^. Meanwhile, according to our national-wide results shown in Table 1, the CO_2_ removal potential by the forest activities in Japan is estimated as an annual average of 175.5 Mt-CO_2_ yr^−1^ by 2042. In this case, forests have the potential to offset 15.3% (175.5/1150) of the national carbon emissions in Japan in the following 20 years, significantly contributing to the net-zero target with the implementation of effective management strategies for existing forests and expansion of forestation. When considering three subcategories, local cities hold the highest potential of CO_2_ removal in total for the 25 years compared to megacities and regional cores accounting for 83.8% of the national total amount (see supplementary Table S1 for details).

**Table 1:** Sustainable and forest-CO_2_ indicators in different regions.

		Land Area	Forest Area	CO_2_ Removal	CO_2_ Value	NC	IW	GDP
		(Million ha)		(Mt-CO_2_ yr^−1^)	(Billion US$)			
Geographical Regions	Hokkaido	7.84	5.20	26.5 ± 2.19	2.32 ± 0.19	60.9	2261.9	176.0
	Tohoku	6.70	4.46	32.78 ± 1.81	3.56 ± 0.20	45.4	3882.9	307.4
	Kanto	3.24	1.32	10.14 ± 0.51	0.9 ± 0.05	16.9	20,159.0	1965.6
	Chubu	6.68	4.32	30.86 ± 1.71	2.91 ± 0.16	42.3	9863.1	901.5
	Kansai	3.31	2.12	18.27 ± 0.77	1.73 ± 0.07	19.8	10,169.3	846.7
	Chugoku	3.19	2.23	21.35 ± 0.86	1.97 ± 0.08	20.0	3211.6	271.6
	Shikoku	1.88	1.35	11.93 ± 0.49	1.18 ± 0.05	12.0	1611.8	129.3
	Kyushu	4.45	2.58	23.71 ± 1.00	2.21 ± 0.09	28.0	5718.1	466.9
Cite Types	Megacities	3.37	1.55	12.95 ± 0.38	1.23 ± 0.04	16.1	28,419.0	2687.1
	Regional Cores	3.60	2.20	15.54 ± 0.86	1.27 ± 0.07	24.3	4745.4	401.8
	Local Cities	30.33	19.83	147.04 ± 8.1	14.28 ± 0.79	204.9	23,713.3	1976.0
Total of Japan		37.30	23.59	175.53 ± 9.34	16.78 ± 0.89	245.3	56,877.7	5065.0

The maps for the 8 geographical regions are shown in supplementary Fig. S2. Supplementary Table S1 also presents the regional proportions for each indicator. CO_2_ Removal and CO_2_ Value are presented with a 95% confidence interval (CI). CO_2_ Value is measured as the annual average value.

The results of forest CO_2_ removal differ according to different data sources and forest growth models. It is reported that the annual net C-uptake in Japan’s forests from 1966 to 2012 was 19.9 TgC yr^−1^ using volume data from the NFI, which was largely underestimated because volume-age relationship parameters used in the NFI were outdated^38^. This study updated the parameters with the latest data, we estimated a higher annual C-uptake value at 32.8 TgC yr^−1^ between 2007 and 2012. Iizuka and Tateishi^39^ estimated the total forest CO_2_ sequestration in Japan is 111.27 Mt-CO_2_ yr^−1^ with multiple RS datasets, which is lower than our projection of 175.5 Mt-CO_2_ yr^−1^. One reason is that they didn’t count the potential from afforestation; the other reason is that they used a national-uniform forest volume growth model, which may underestimate the sequestration capacity in some regions. Therefore, our cross-examination of multiple data sources and localization of model parameters could improve the accuracy of CO_2_ removal estimation.

As for the temporal trend, the yearly potential of CO_2_ removal by FM and AR as a total will reach the highest around the year 2027 to 2030 in all four categories. After the year 2030, CO_2_ removal potential will begin to decrease, and it will decline faster in megacities than in other areas. The potential from managing existing forests will peak near 2030 and then decrease after 2030; while the potential from afforestation will continuously increase but play a minor role, especially in the beginning years. Furthermore, the higher the AR area will be, the higher the total CO_2_ removal will be, and the slower the downward trend of total removal after 2030 will be. This is due to differences in the carbon sequestration capacity of forests of different ages. After the plantation, the growth rate of forest vegetation follows the sequence slow–vigorous–slow–stop^44,45^ (see supplementary Figs. S3, S4). This means aging trees (> 50 years) have a lower potential to sequestrate and remove CO_2_ than young trees. Therefore, afforestation increases the proportion of young and fast-growing trees and can offset the decreasing trend of potential from existing forests in later years. After 2030, effective tending management such as selective cutting, thinning, and regeneration is also required to maintain the high carbon sequestration level of existing forests.

**Figure 2:**
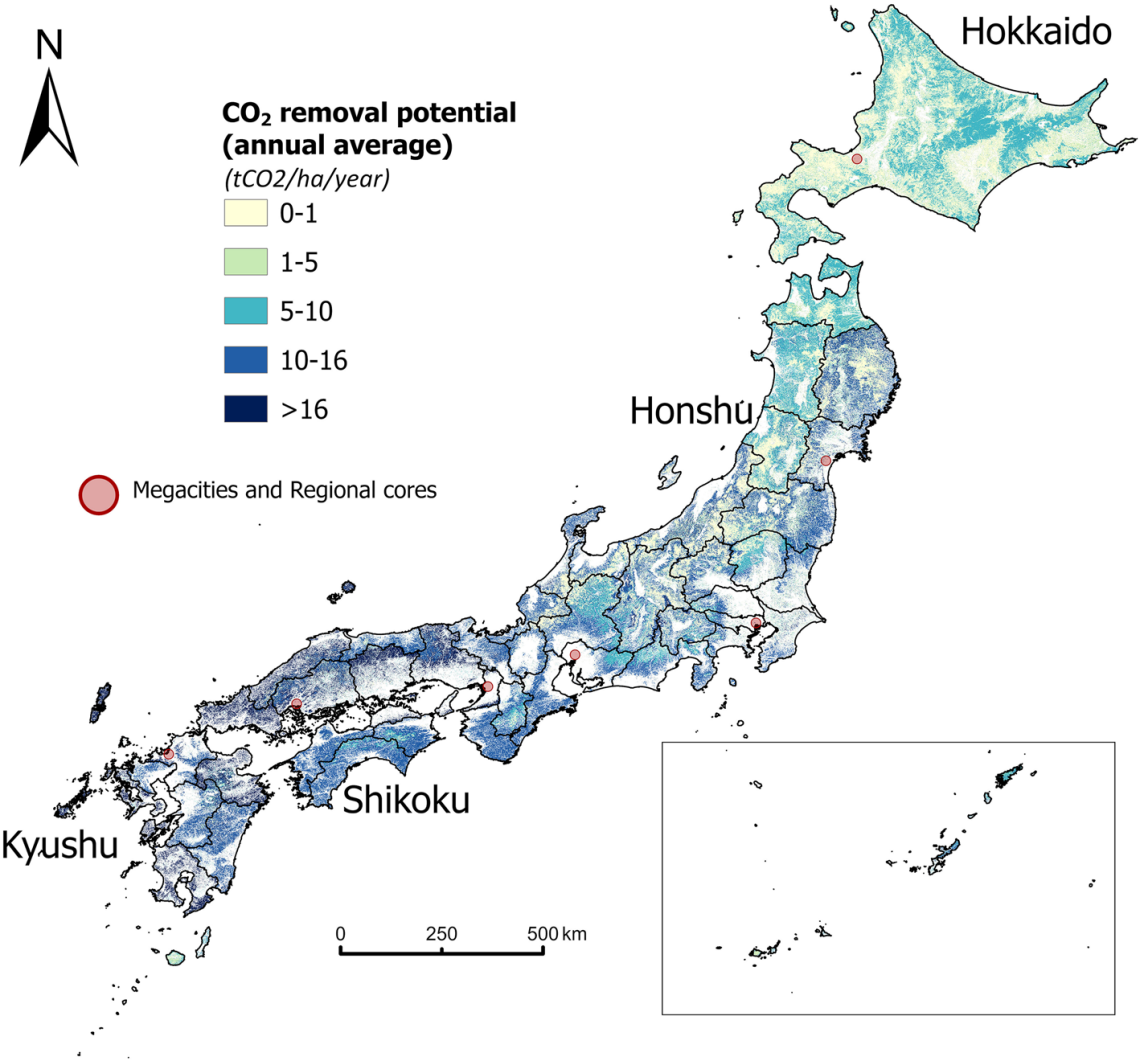
Spatial distribution of CO_2_ removal potential of existing forests at the pixel level. The map was generated with ArcGIS Pro 3.1.3 (https://pro.arcgis.com/en/pro-app/3.1/get-started/get-started.htm). The four main islands of Japan (Hokkaido, Honshu, Shikoku, Kyushu) are marked on this map.

Figure 2 presents the spatial pattern of the per-hectare potential of CO_2_ removal (annual average from 2018 to 2042) for existing forests at the pixel level (approximately 1 hectare). The annual CO_2_ sequestration rate in Japan shows a roughly decreasing pattern from south to north. The spatial distribution of the CO_2_ removal potential from all the existing forests indicates that forests in the south have stronger capacities to sequester CO_2_ than in northern regions which is consistent with the pixel-level results of Iizuka and Tateishi^39^, who used multiple satellite data. Specifically, the per hectare CO_2_ removal in Hokkaido is lower than in other regions because the age structure of forests in Hokkaido is old, and the major planted type is pine, which grows slower than the major planted species (Sugi and Hinoki) in other regions (see histograms for age classes and tree species in supplementary Figs. S5, S6). The urban–rural difference is also significant. It shows that the potential of CO_2_ removal from forests is trivial in those major cities, especially for megacities. In contrast, remote areas including the mountainous areas in southwestern Honshu and southern Shikoku show the highest potential value of CO_2_ removal from forests. Meanwhile, the forest CO_2_ removal potential (averaged by forest area) in northern Japan, especially in Hokkaido is relatively low.

### Market potential of forest CO_2_ removal in monetary value

**Figure 3:**
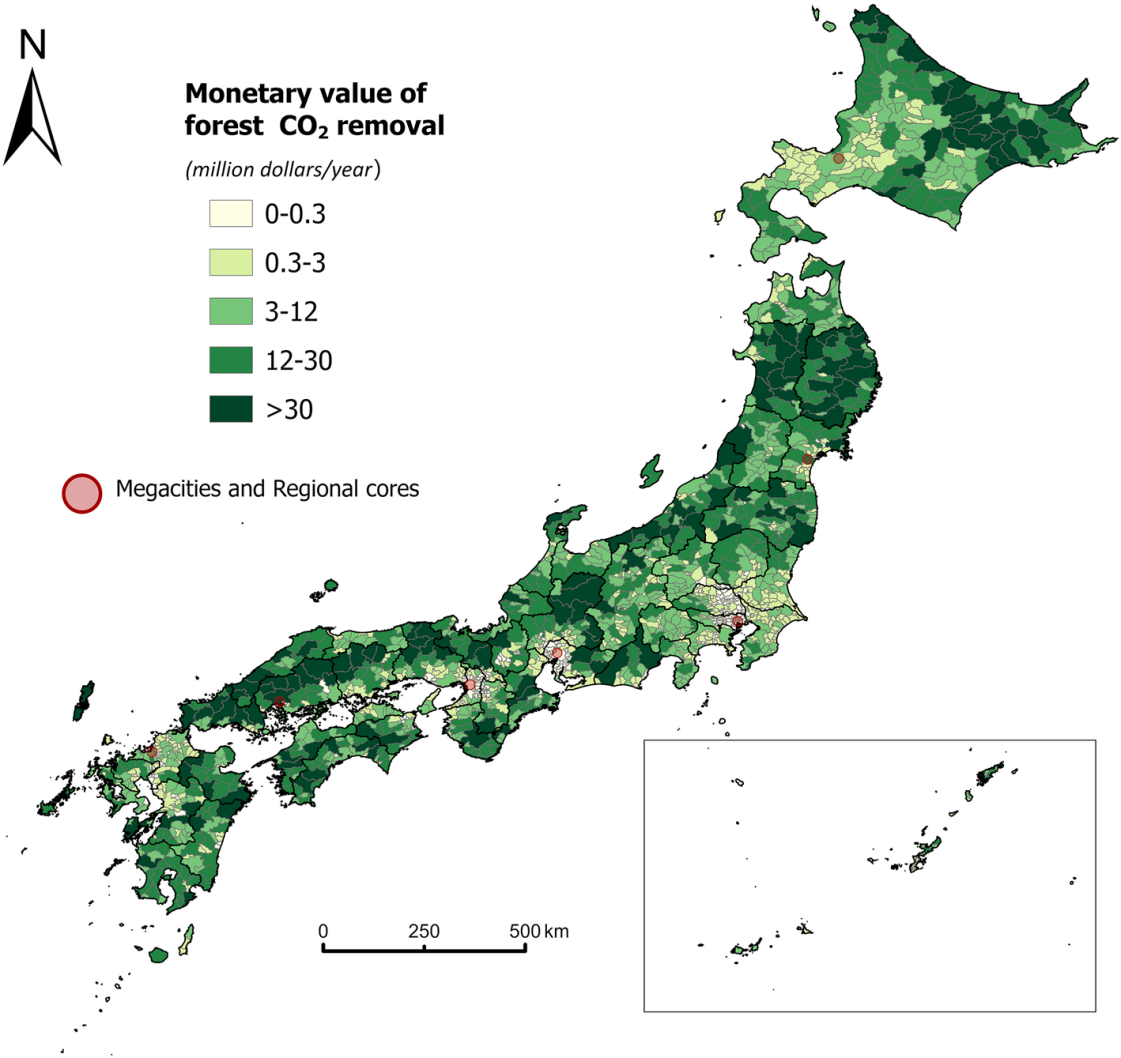
The monetary value of annual average CO_2_ removal by forests at the municipal level. The map was generated with ArcGIS Pro 3.1.3 (https://pro.arcgis.com/en/pro-app/3.1/get-started/get-started.htm).

Figure 3 presents the 25-year-average annual monetary value of forests in terms of CO_2_ removal potential by FM and AR at the municipal level. Although restricted forest areas contribute to CO_2_ removal, they are hardly used for the carbon credit market due to the strict limitations on human activities there (see supplementary Fig. S7 for the extent of restricted forest areas). Therefore, only managed forests are measured for CO_2_ monetary valuation with a fixed price in the J-credit market. In this context, for a given region, other factors being equal, we can assume that the higher the monetary value of forest CO_2_ removal, the higher the number of carbon credits possibly available for market trading, and the higher the market potential.

Compared with the pixel-level CO2 removal amount as Fig. 2 shows, the north–south disparity in monetary value (Fig. 3) is not as significant. Because in addition to the carbon sequestration capacity per unit area, the monetary value in a municipality is also influenced by the area of forest available for market trading. For example, although the pixel-level forest CO_2_ removal (t-CO_2_/ha) is low within some municipalities of northeastern Hokkaido (Fig. 2), the total monetary value for the whole municipality is at the highest level (above 30 million dollars per year) due to the vast forest cover in there (Fig. 3).

Though the north–south variation is not clear for the monetary value of forest CO_2_, the difference in monetary value between urban and rural communities is evident. Figure 3 clearly shows that cities in rural hilly areas (northeastern Hokkaido, northern Honshu, and the inner areas of western Japan) have much higher market potential for forest CO_2_ removal than megacities and regional cores. It indicates that rural local communities have the potential to create new value-added in these cities by FM and AR.

### Value-added to sustainable development

We calculated the municipal-level IW and NC of Japan for the years 2010 and 2017. The total NC of Japan in 2017 is estimated at 245.3 billion US$; meanwhile, the annual value of forest CO_2_ removal in the future by 2042 is 16.8 ± 0.89 billion US$ (Table 1). In other words, if the CO_2_ removal value is counted in the forest capital valuation, it will contribute 7% to the annual increase of NC in Japan. In some rural municipalities, the contribution rate can reach more than 12% (see the NC contribution map at the municipal level in supplementary Fig. S8).

**Figure 4:**
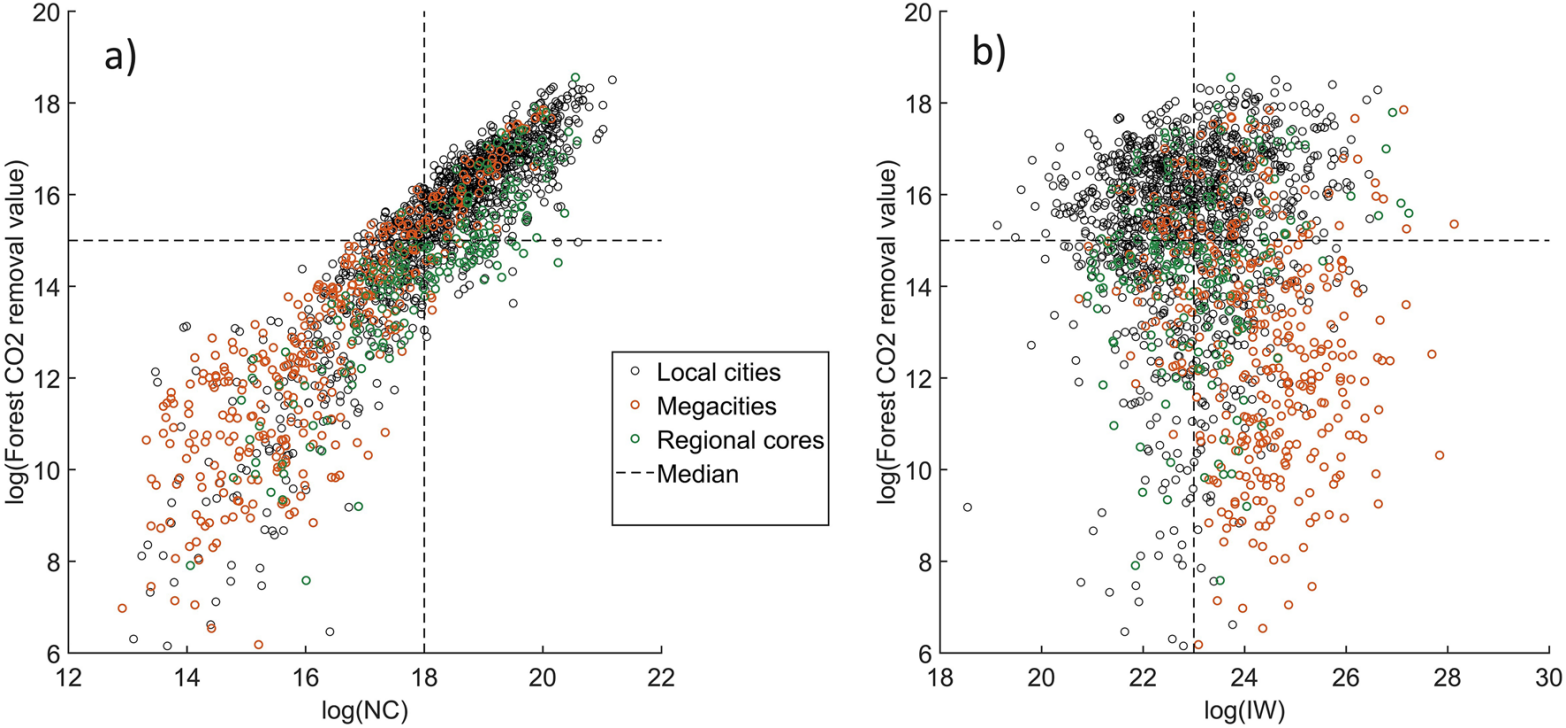
The relationship between forest CO_2_ removal potential and (**a**) NC, (**b**) IW. The figure was generated with MATLAB 2023a (https://ch.mathworks.com/products/matlab.html). The vertical dashed lines indicate the median of NC and IW, and the horizontal dashed lines indicate the median of the market potential of CO_2_ removal for 1743 municipals of Japan.

We further explored the relationship between forest CO2 removal and NC and IW as shown in Fig. 4. Using the median as a threshold, the data were classified into high values and low values. There is a positive relationship between forest CO_2_ removal and NC (Fig. 4a) as forest is a major component in natural capital. Most megacities are in the left-bottom of the figure with a relatively low value in both NC and forest CO_2_ removal potential. In contrast, most local cities are at the right top of the figure indicating being richer in both NC and forest CO_2_ removal potential. On the other hand, the relationship between forest CO_2_ removal and IW is relatively complicated (Fig. 4b). Megacities have lower forest CO_2_ removal values but higher IW. Local cities usually have a higher potential for forest CO_2_ removal, but not necessarily less IW.

**Figure 5:**
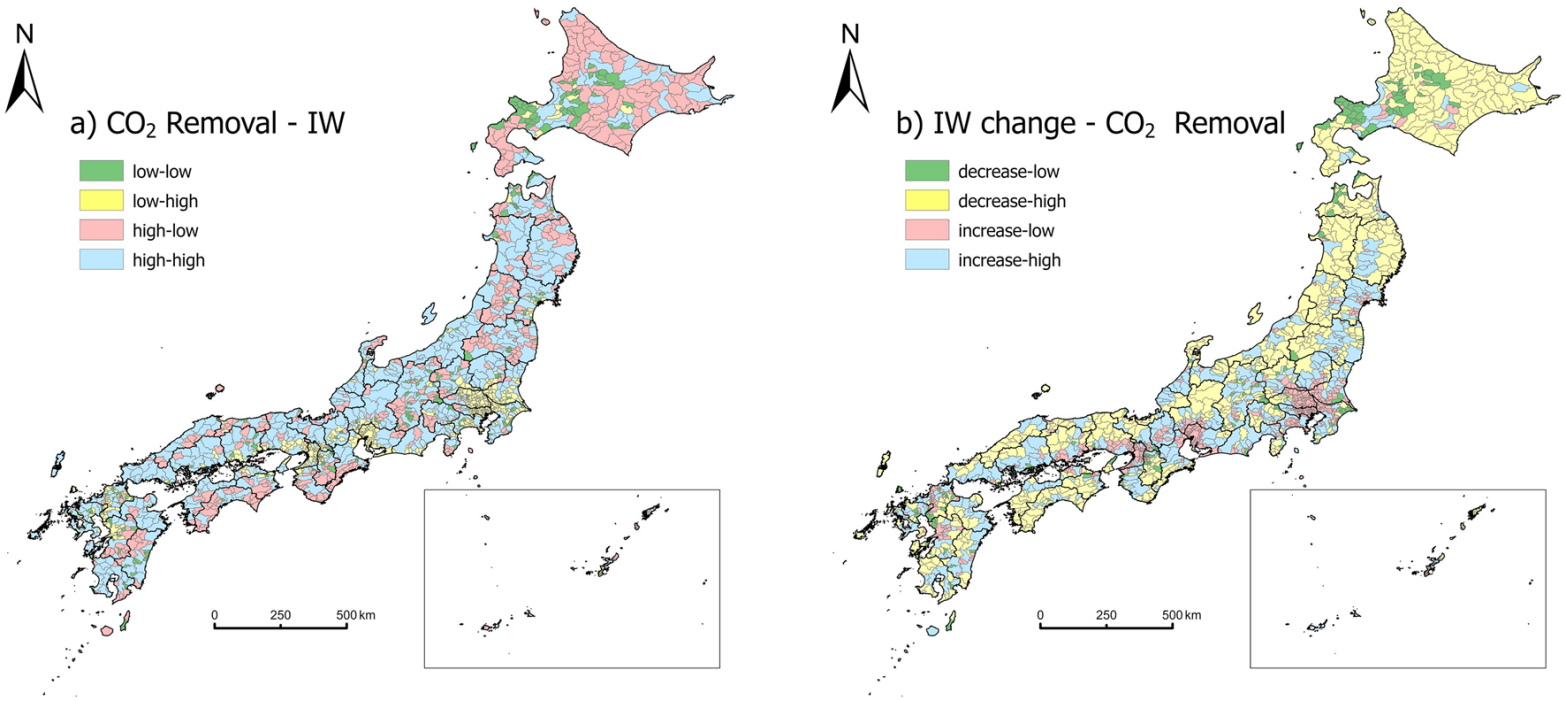
The coupling relationship between forests CO_2_ removal and (**a**) IW, (**b**) IW change. The map was generated with ArcGIS Pro 3.1.3 (https://pro.arcgis.com/en/pro-app/3.1/get-started/get-started.htm). IW change refers to the change of IW between 2010 and 2017.

The municipalities belonging to the four divisions of Fig. 4b are further mapped in Fig. 5a. Municipalities are also classified as sustainable (IW increase) and unsustainable (IW decrease) at different levels of CO_2_ removal (Fig. 5b). Megacities are sustainable with high IW but lower market potential in forest carbon sink. Most unsustainable cities (74% of all unsustainable cities, Fig. 5b) have a higher potential for forest CO_2_. While for the local communities in green colors, special attention should be paid to them. Because IW in these municipals is low and still decreasing, which indicates an unsustainable community; meanwhile, the market value created from forest CO_2_ removal there in the future is also lower than the median level. By our estimates, such communities comprise approximately 10% of the entire Japan.

## Conclusion

The forest sink has been playing a key role in reducing and removing carbon emissions to mitigate climate change^3,32^, especially for a country like Japan, which is rich in forests. However, the spatially explicit projection of its potential to remove CO_2_ and how this will contribute to the development of a sustainable community has never been investigated before. In this context, this study predicts the future potential of CO_2_ removal by forest activities in Japan at the country, prefecture, municipal, and pixel levels, then transfers the amount of forest CO_2_ removal into monetary value based on a national carbon emission trading market. Furthermore, this paper also investigates how the CO_2_ removal value created by forests contributes to sustainable development in big cities and rural communities, by putting it under the framework of inclusive wealth.

According to the national-level analysis, we reinforce the importance of Japan’s forest sink, not only in offsetting the national carbon emission by 15.3% but also in adding value to natural capital and inclusive wealth at a non-negligible level. The temporal analysis suggests a turning point in 2030; after that year, afforestation and effective tending management of existing forests are required to maintain the previous carbon sequestration rate of Japan’s forests. More importantly, with the spatial disaggregation analysis, this study generated important findings regarding the potential of forest CO_2_ removal and how these potentials link to sustainable community development. Our study highlighted the differences between Japan’s rural and urban communities. Wealth growth is spatially uneven and displays an opposite spatial pattern to forest CO_2_. IW values in megacities and regional cores are high and still increasing, while many rural communities are unsustainable in terms of IW growth, with much lower IW value. In contrast, rural communities usually have a much higher potential for forest CO_2_ removal than urban communities. It means that they have an opportunity to increase IW through the market potential of carbon removal, whose value was always ignored or underestimated before. Based on this result, our work suggests that a national carbon emission trading market will benefit the sustainable development of both megacities and rural communities. Urban areas with a growing population and wealth will also generate high carbon emissions^46^. However, the cost of carbon emission in megacities will increase due to the purchase of emission rights such as J-credits from forest sinks. This encourages them to transfer to environmentally friendly behaviors to save energy and reduce emissions, which contributes to a sustainable community. On the other hand, the trading of forest CO_2_ credits within the country can promote the transfer of income to rural areas which would affect to increase in human well-being in those communities through public investment.

Though the market potential of forest sinks is significant, currently only a minor proportion of them has been available on the carbon emission trading market. According to the certification data from the J-credit market^47^, only 0.27 t-CO_2_ from the forest sink was verified as credits, and only 20% of those credits could be sold. One possible reason for this is the lack of transparent information about credit creators and forest owners^47^. Therefore, companies or groups aiming to reduce emissions from forest carbon offset projects (credit buyers) could benefit from our spatially explicit data of forest CO_2_ removal to find potential credit creators. Furthermore, governments that are interested in spatially priority implementation and tracking of local forest mitigation targets could also make use of such data. For example, our results highlight some rural communities of inland Japan with decreasing IW and low CO_2_ removal potential. Policies in these regions should prioritize seeking new added value beyond the forest carbon trading market.

Still, there are limitations to be noted for further investigation. First, forests provide a range of goods and services that are vital for human well-being and economic development, as well as the sustainability of communities, especially for rural communities that are reliant on forests for income and subsistence^28^. However, only the service of CO_2_ removal from forests can be valued, certificated, and traded in the J-credit market now. In order not to underestimate the importance of forests in sustainable communities, a more comprehensive valuation for forests like natural capital accounting in the IW framework is expected to be introduced in the credit-trading system in the future. Secondly, the spatial prediction of forest carbon sequestration is highly subject to data availability^48,49^. However, the data of yielding parameters to predict tree growth are not available for every prefecture of Japan; and we only consider the CO_2_ removal by forest vegetation, without counting other forest carbon pools like the soil due to the lack of data and clear certification criteria in Japan. With more high-resolution monitoring data for forest growth and carbon absorbing becoming available in the near future, compiling and mapping more accurate, timely, and cost-effective carbon accounts will become increasingly feasible. Furthermore, the market potential of forest CO_2_ removal and its contribution to sustainable communities could be influenced by various factors such as carbon price fluctuation, climate change and natural disturbances, and social networks and residents’ behaviors in the communities^3,27,28^. Therefore, further investigation should take care of how those factors will impact the role of forests in mitigating climate change, as well as the potential impacts on forest-dependent communities.

## Methodology

### Calculation design and scope

We predicted Japan’s future forest CO_2_ removal at the prefectural level with the NFI data, then spatially downscaled the estimation to the pixel level with spatial data products from remote sensing, and also aggregated the pixel value to the municipality level excluding reserve and restricted forests that cannot be certified in the J-credit system. Furthermore, using the shadow price for forest CO_2_ derived from the J-credit trading data, we obtained the monetary value of forest CO_2_ removal for each municipality of Japan and compared it with natural capital and inclusive wealth in terms of sustainable development. We also included a flowchart for this in supplementary Fig. S9.

Since the latest NFI data is for the year 2017, the period of projection includes 25 years from 2018 to 2042. The temporal trending of forest CO_2_ removal change was analyzed at levels above prefectures. To show the spatial variety, results at the pixel- and municipality-levels were mapped with the 25-years-annual-average value.

When estimating the forest CO_2_ removal, to the extent possible, we adhered to the J-credit guidelines^50^ on the certification of the amount of forest CO_2_ removal, which is adapted from the IPCC Guidelines. Under this framework, forest CO_2_ removal is basically calculated according to forest biomass change, which is further derived from forest volume change. Therefore, to project prefecture-level removal, we first applied a forest volume growth model to relationship data between tree age and tree volume or its annual growth, for different tree species and geographic regions; then we converted the volume change data to the amount of CO_2_ removed from the atmosphere with a forest carbon sequestration model. In addition, being consistent with the IPCC Tier 1 assumption of no net change to non-biomass pools (such as soil) in forest land remaining forest land^34,51^, the J-credit considers only the biomass pool in their certification of forest management activities. Accordingly, we did not include the soil carbon in the forest CO_2_ removal calculation either.

### Data

Four types of datasets were used in this study: prefectural-level NFI data, spatial polygon data of forests and administrative units, gridded land cover data, and gridded forest biomass data.

Firstly, NFI data include forest area (*ha*) data for 12 forest types with 20 age classes covering all the prefectures in Japan (https://www.rinya.maff.go.jp/j/keikaku/genkyou/index1.html). Its latest update was in 2017. The 12 forest types can be mainly divided into two major types: plantation and natural forest. The former includes 10 types: Cryptomeria japonica (P-CJ, or Sugi), Chamaecyparis obtuse (P-CO, or Hinoki), Pinus densiflora (P-PD), Larix leptolepis (P-LL), Abies forests (P-AF), Picea jezoensis (P-PJ), other needleleaf plantations (P-ONF), Quercus actissima (P-QA), Fagus forests (P-FF) and other broadleaf plantations (P-OBF)). Natural forest includes needleleaf forests (N-NF) and broadleaf forests (N-BF). The 20 age classes are summarized in 5-year interval with the first age class being the first to the fifth year and the 20^th^ age class being years over 95.

Secondly, we collected polygon data to identify the spatial extent of nationally owned forests, privately owned forests, as well as reserve forests with strict restrictions on managing activities from the Ministry of Land, Infrastructure Transport, and Tourism of Japan (MLIT) (https://nlftp.mlit.go.jp/ksj/index.html). See supplementary Fig. S7 to identify the distribution of managed and restricted forests in Japan. Besides, the polygon data of Japan’s prefectures and local municipalities (cities, wards, and villages) were also obtained from the MLIT.

Thirdly, we obtained the land use and land change data products from the Japan Aerospace Exploration Agency (JAXA) in three versions (ver.16.09, ver.18.03, and ver.21.11), representing the land use at the pixel level for three time points: 2010, 2015 and 2020 (https://www.eorc.jaxa.jp/ALOS/en/dataset/lulc_e.htm). There are 10 common land use categories in the three versions: water bodies, built-up, paddy field, cropland, grassland, DBF (deciduous broad-leaf forest), DNF (deciduous needle-leaf forest), EBF (evergreen broad-leaf forest), ENF (evergreen needle-leaf forest), and bare. But the spatial resolutions are not consistent for the three versions (10 m, 30 m, and 30 m, respectively). Therefore, we resampled the 10 m-resolution data to 30 m-resolution and generated the maps of land conversion types between different years.

Lastly, we also created a national forest biomass map using high-resolution data obtained from Global Forest Watch (GFW) (https://www.globalforestwatch.org/map/), which provides the aboveground live woody biomass (AGB) density (Mg/ha) with 30 m-resolution.

### Forest volume growth model

The forest volume growth model estimates the relationship between tree age and stem volume. Yield tables can provide the empirical results for this relationship at stand-scale for a specific forest type in a specific region under a specific management regime, and are widely used in forest CO_2_ removal estimation^38^. However, most yield tables have not been published, leading to the lack of volume growth data in most prefectures. And most published ones were constructed before the 1980s, which are outdated. In this study, we integrated all recently updated yield tables (after 2010) available for Japan’s regions and constructed the forest age-volume relationship using three different forest volume growth models. The three procedures are presented as follows.

1. For Sugi (P-CJ) and Hinoki (P-CO)

P-CJ and P-CO are the most important planted tree species in Japan, occupying 70% of Japan’s planted forest area by our estimates. Governments have developed specific standardized management plans for these two forest types, and normally provide the Stand Density Management Diagram (SDMD) in the forestry plans to control management measures^52,53^. Based on the SDMD, the stem volume can be estimated with the number of trees and the stand height as below^54,55^:

1 $${V}_{Rf}={({a}_{1}\cdot {H}^{{b}_{1}}+{a}_{2}\cdot {H}^{{b}_{2}}/{N}_{Rf})}^{-1}$$

where ${V}_{Rf}$—the stem volume at ${N}_{Rf}$ (m^3^/ha); ${N}_{Rf}$—the maximum stand density (trees/ha); $H$—the stand height (m); ${a}_{1}$, ${a}_{2}$, ${b}_{1}$, ${b}_{2}$—constants for the equation obtained from local SDMDs (see their values for specific regions and tree species in supplementary Table S2).

${N}_{Rf}$ has a relationship with $H$ as below:

2 $${{\text{log}}(N}_{Rf})={c}_{1}+{c}_{2}{\text{log}}(H)$$

where ${c}_{1}$, ${c}_{2}$,—constants for the equation obtained from local SDMDs (see their values for specific regions and tree species in supplementary Table S2).

The stand height $H$ (m) at different stand age $t$ can be obtained by empirical height growth curves^53^. Based on the meta-analysis of the local yield tables of Japan, we used two major forms for the height growth curve in this study^56–59^.

3 $$H=\alpha \cdot (1-\beta \cdot {\text{exp}}\left(-\gamma \cdot t\right))$$

or

4 $$H=\alpha \cdot {(1-{\text{exp}}\left(-\beta \cdot t\right))}^{\gamma }$$

where $\alpha$, $\beta$, $\gamma$—equation parameters obtained by fitting observed data (see their values for specific regions and tree species in supplementary Table S3).

The stem volume ${V}_{s}$ can be calculated as below:

5 $${V}_{s}={{V}_{Rf}\cdot R}_{y}$$

where ${R}_{y}$ is the yield ratio, an indicator of relative forest density. In forestry practice, ${R}_{y}$ should be managed at a proper level. For example, to avoid delayed thinning, ${R}_{y}$ should not exceed 0.85 or 0.80. In this study, we assume ${R}_{y}$ at 0.7 as most regional forest plans in Japan suggest.

(2) Pine forests

Four pine tree species are counted in the NFI (P-PD, P-LL, P-AF, P-PJ). The stand height for them was also calculated by Eq. (3)^60^. Due to the absence of SDMD for pine forests, we estimated the stem volume for pine forests by the Schumacher-Hall equation as below^61,62^:

6 $${{\text{ln}}(V}_{s})={p}_{1}+{p}_{2}\cdot {\text{ln}}\left(DBH\right)+{p}_{3}\cdot {\text{ln}}\left(H\right)$$

where $DBH$—the mean diameter at breast height (cm); ${p}_{1}$, ${p}_{2}$, ${p}_{3}$—equation parameters obtained by fitting observed data (see their values in supplementary Table S4).

$DBH$ is calculated by using an annual growth rate ${r}_{d}$ of DBH^60^:

7 $${r}_{d}=m\cdot {\text{exp}}\left(-n\cdot t\right)$$

where $m$, $n$—constants for the equation (see their values in supplementary Table S4).

(3) Broadleaf forests

Due to the mixed composition in broadleaf forests, it is hard to model the volume growth by explicit equations. Therefore, we used the empirical observation data for three tree broadleaf types from the yield table published by the local government of Gifu^63^.

Based on Eqs. (1)-(7), the relationship curves between stem volume and stand age for eight tree types in eight regions of Japan are presented in supplementary Fig. S3.

### Forest carbon sequestration model

According to the guidelines of J-credit and IPCC, the carbon stock of forests ${C}_{B}$ in a certain region is calculated as the sum of above-ground and below-ground biomass (*t-C*)^50,51^:

8 $${C}_{B}=\sum_{i,j}{A}_{i,j}\times {V}_{i,j}\times {WD}_{i}\times {BEF}_{i,j}\times CF\times \left(1+{R}_{i}\right)$$

where $i$—tree species/forest type; $j$—tree stand age; $A$—the area of land remaining in the same land-use category, (ha); $V$—the stem stock volume (m^3^/ha); $WD$—the basic wood density (t-dm/m^3^); $BEF$, the biomass expansion factor; $CF$—the carbon fraction of dry matter which is set as 0.5 t-C/t-dm; $R$—the ratio of below-ground biomass to above-ground biomass.

In Eq. (8), the constants of *WD, BEF,* and *R* for specific tree species, stand ages, and geographic regions in Japan are obtained from Table 3-2 in Japan’s Supplementary Information on LULUCF Activities under Kyoto Protocol 3.3 and 4 (SI-KP3.3&4) (https://www.env.go.jp/content/900447700.pdf). The volume $V$ for specific forest type and age were obtained through the forest volume growth model.

For future forest area ${A}_{i,j}$, we consider the data for AR and FM separately: (1) for FM, we assume existing forests remain forest in the future, and the forest age increases year by year over time. (2) for AR, we assume the afforestation area is in proportion to the existing forest area for all prefectures, and the AR rates are nationally uniform. But we set three scenarios for the future AR rate: 3.5%, 2.1%, and 0.7% every five years. For the highest rate, it comes from historical land cover data, because according to our calculation with the JAXA data, the rate of land conversion from other lands to forests over the past five years is 3.5%. The middle value was set based on the latest National Forestry Plan^30^, suggesting the ratio of the target afforestation area in the next five years to the present forest area is approximately 2.1%. Therefore, we set three scenarios and analyzed how different AR amounts will affect the total CO_2_ removal potential. The area proportion of tree species in new forests in each prefecture is also the same as that in existing forests.

Therefore, the CO_2_ amount removed by forests during two time points ${t}_{1}$ and ${t}_{2}$ can be estimated through the carbon stock change^50^:

9 $$\Delta {C}_{RE}=(\Delta {C}_{BC}-\Delta {C}_{LC})\cdot 44/12$$

10 $$\Delta {C}_{BC}=({C}_{{B}_{2}}-{C}_{{B}_{1}})/({t}_{2}-{t}_{1})$$

where $\Delta {C}_{RE}$—the CO_2_ removal amount between ${t}_{1}$ and ${t}_{2}$ (t-CO_2_); $\Delta {C}_{BC}$—the carbon stock change in forest biomass; $\Delta {C}_{LC}$—the carbon stock loss due to the land use conversion; ${C}_{{B}_{2}}$, ${C}_{{B}_{1}}$—the carbon stock in forest biomass at ${t}_{1}$ and ${t}_{2}$. For CO_2_ removal potential from FM, we assume the existing forest will remain as forests in the future, $\Delta {C}_{LC}$ is set as zero in this study. For CO_2_ removal potential from AR, based on the SI-KP3.3&4, if the lands for afforestation are converted from grasslands, $\Delta {C}_{LC}$ is assumed as 6.75 t-C/ha multiplied by the land conversion area; if the lands are converted from croplands or other land-use types, $\Delta {C}_{LC}$ is set as zero. The future AR area transformed from grasslands is estimated by the percentage of the forests converted from grasslands in the forests converted from all other land-use types using the JAXA land cover data.

Using the forest volume growth and carbon sequestration model, we estimated the forest carbon stock in 2007, 2012, and 2017 and projected the amount of CO_2_ removal from both existing forests and afforestation from 2018 to 2042 at the prefectural level of Japan.

### Downscaling CO_2_ removal potential to pixel level

Based on the forest volume growth model, we also got the relationship between stand age and annual volume growth as supplementary Fig. S4 shows. Accordingly, the growth rate of stem volume usually increases first and then decreases as the tree ages. This means that the future CO_2_ removal potential and the present forest biomass do not follow a simply linear-positive relationship (supplementary Fig. S10).

Though we obtained the volume-age relationship for eight tree species by the above procedures, only three types of forests can be easily identified: ENF, DNF, and BF (broad-leaf forests) at the pixel level. Therefore, we further categorized the eight species into ENF, DNF, and BF and calculated the average volume at each stand age for the tree species in each category of the three forest types. Converting the average volume to forest biomass by Eq. (8), we got the forest biomass over stand age for the three forest types. Therefore, we could obtain the pixel-level tree age maps for both historical and future years based on this relationship with the JAXA land cover data and the GFW forest biomass data (see supplementary Fig. S11) as Izuka and Takeishi did^39^. Furthermore, the relationship between forest CO_2_ removal amount and stand age for the three forest types was also obtained by Eq. (9). Accordingly, we obtained the pixel-level forest CO_2_ removal maps by combining them with the tree age maps.

To note, we also adjusted the pixel-level value by multiplying a prefecture-specific factor to ensure the sum of pixel values for each prefecture in this step equals the prefectural value estimated based on NFI data. Lastly, we built a CO_2_ removal potential map (25-year annual average) at the pixel level for the existing forests in Japan.

### Monetary value of forest CO_2_ removal at the municipal level

On one hand, the municipal level CO_2_ removal amount from managing existing forests could be calculated by summing the pixel values within it. However, not all forest CO_2_ removal could be certified as FM credits and traded in the J-credit market^50^. In this study, we excluded the CO_2_ removal of the reserve and restricted forests from the total. These forests are mainly maintained by natural regeneration, and their primary function is not for wood production and economic benefits. On the other hand, the municipal level CO_2_ removal from AR was calculated by allocating the prefecture-level AR removal to each city in proportion to its forest area. Thus, the monetary value of forest CO_2_ removal at the municipal level was calculated by multiplying the market price of forest CO_2_ and the total amount of CO_2_ removal from AR and FM. The market price is assumed as 14,650 JPY/ t-CO_2_, which is the average price of the credits from forest sinks based on the J-credit market demonstration conducted by Japan Exchange Group from September 2022 to January 2023^6^.

### Inclusive wealth

The IW approach measures the multitude of capital assets in an economy and the weighted sum of the stocks of those assets. The weights are the marginal contributions of the stocks to intergenerational wellbeing, also called the assets’ shadow prices, and the weighted sum is called the economy’s inclusive wealth. In a practical setting, IW measures the monetary value of three types of capital assets: human capital (HC), produced capital (PC), and natural capital (NC). IW can be formulated as follows^13^:

11 $${\text{IW}}={P}_{HC}\cdot {K}_{HC}+{P}_{PC}\cdot {K}_{PC}{ + P}_{NC}\cdot {K}_{NC}$$

where ${K}_{HC}$, ${K}_{PC},$ and ${K}_{NC}$ denote the stocks of HC, PC, and NC, respectively. ${P}_{HC}$,${P}_{PC}$, ${P}_{NC}$ denote the shadow prices of HC, PC, and NC, respectively. Increases in IW indicate an improved productive base capable of supporting a higher standard of living in the future consistent with sustainable development, whereas decreases in IW indicate unsustainable development.

Specifically, human capital (HC) is embodied in people in terms of education, skills, health, etc.; produced capital (PC) is also called physical capital and includes manufactured assets such as infrastructures, buildings, and machinery; and NC comprises nonrenewable resources (fossil fuel and minerals) and renewable resources (the services and products provided by ecosystems). In this study, we also calculated the inclusive wealth in 2010 and 2017 at the municipal and gridded level, for the comparison analysis for forest carbon sequestration and sustainable development. The calculation procedure strictly followed the materials and methods proposed by Zhang et al.^9^, we will not go into that too much here.

However, we still present how the natural capital was calculated as it is one of the most important indicators of interest in our study. Since the nonrenewable natural resources are scarce to be negligible in Japan^16^, only renewable resources were involved here. The wealth of renewable resources includes the wealth from ecosystem products and services from six main biome types in line with six land cover types, including forests, water bodies, croplands, mangroves, wetlands, and grasslands. The wealth of each biome at grid cell *j* for year *t*,${ESW}_{jt}$, can be represented as follows^8,9^:

12 $${ESW}_{jt}=\sum_{i}\left({WP}_{it}\cdot {Q}_{ijt}\right)$$

13 $${WP}_{it}={\int }_{0}^{\infty }\left({P}_{it}\cdot {r}_{it}\cdot {e}^{-\delta \cdot s}\right)ds$$

In Eq. (12), *t* is the year under analysis, which is 2010 or 2017 in this study; ${Q}_{ijt}$ is the total area of biome *i* at grid cell* j*; and ${WP}_{it}$ can be regarded as the shadow price of biome *i*. In Eq. (13), $\delta$ is the discount rate, here assumed to be fixed at 5%, and ${r}_{it}$ is the fraction of the total area of ecosystem *i* that is accessed by individuals to obtain benefits, here assumed to be 10%^16^. ${P}_{it}$ is the flow value of ecosystem services of biome *i* in monetary units.

In this study, we used a benefit transfer method to obtain ${P}_{it}$ for each biome with the Ecosystem Service Valuation Database (ESVD) following the procedure presented by de Groot et al. (2012)^64^. To note, for forests, the services of provisioning, regulating, culture, and habitat were valued in monetary units. However, the regulating service of climate was valued as zero in that procedure.

### Uncertainty analysis

Previous studies^39,51^ show that three main factors that could generate uncertainties in the results include: the accuracy of input data, the age-volume relationship, and the sequestration parameters. Accordingly, in this study, we have already attempted to minimize the uncertainties pointed out by existing studies and improve the estimation models in the following ways: (1) We used the highest-resolution and the highest-accuracy land cover and forest biomass data available to minimize the uncertainties from pixel-level spatial data. (2) We also cross-examined the pixel-level data and prefectural statistical data, to ensure that the sum of pixel value is equal to the corresponding statistical value when aggregating pixels to administrative units. (3) In the forest volume growth model, we localized the parameters that are specific for different regions, forest types, and tree ages with the latest data sources. This helps to increase the precision and accuracy compared with previous studies, which used national-standardized parameters for the whole of Japan; and we also used localized (IPCC Tier 3) sequestration parameters to convert volume to CO_2_ removal.

In addition to that, we also conducted sensitivity analyses for two variants that have been recognized as crucial factors in the forest volume growth and carbon sequestration model^39,51^ (see supplementary Table S5 for the results):

Variant 1: sensitivity to land productivity.

The yield tables also show empirical relationships under different land-productivity indices (stand sites for trees). Higher land productivity leads to higher growth rate of forest volume. Normally, in the local yield table construction system (LYCS) of Japan^59^, the forest areas are classified into three types by land productivity. The three types are denoted as site S: 1, 2, and 3, representing high, average, and low growth rate areas. However, no clear standard exists on how the land productivity index or stand site is determined, which is a large source of error for stem volume estimation^38^. In empirical studies of LYCS, the parameter $\alpha$ in Eqs. (3) and (4) has a negative linear relationship with site S, meaning a positive relationship with land productivity. The higher the value of parameter $\alpha$, the better stand conditions, and the higher and faster growth of trees^56^. Therefore, we changed the value for parameter $\alpha$ and showed how the change influences the results.

Variant 2: sensitivity to assumptions on the pre-afforestation land use.

In the standard model of this study, afforestation lands are assumed to be converted from either grasslands or other lands. The former implies that there are carbon emissions during afforestation, while the latter does not. The grassland conversion area was projected with a historical conversion rate. Here, we showed how the results change if all the pre-afforestation lands are grasslands or none of them are grasslands.

Furthermore, following the method presented in Egusa (2020)^38^, we calculated the 95% confidence interval (CI) for annual average CO_2_ removal projections, to show the degree of variation of results.

### Supplementary Information

Supplementary Information.

## Data Availability

The data used for this study are either publicly available (see Methodology section) or can be found in the Supplementary Information.
