# Supplementary material for: Forest carbon removal potential and sustainable development in Japan
Source: Sci Rep. 2024 Jan 5;14:647. doi: 10.1038/s41598-024-51308-z (PMC10770060; doi:10.1038/s41598-024-51308-z)
Supplement: Supplementary file 1 — Supplementary Information. [file 41598_2024_51308_MOESM1_ESM.pdf]

Supplementary Material for

Forest carbon removal potential and sustainable development in Japan

Bingqi Zhang, Janaki Imbulana Arachchi, Shunsuke Managi

Shunsuke Managi

Email: managi @doc.kyushu-u.ac.jp

This PDF file includes:

- **Tables S1 to S5**
- **References for Tables S2 to S4**
- **Figs. S1 to S11**

Note:

Figs. S1-S2, Figs S7-S8, and Fig. S11 were generated with ArcGIS Pro 3.1.3 (https://pro.arcgis.com/en/pro-app/3.1/get-started/get-started.htm);

Figs. S3-S6 and Fig. S10 were generated with MATLAB 2023a (https://ch.mathworks.com/products/matlab.html);

Fig. S9 was generated in PowerPoint ver.2302 (https://www.microsoft.com/en-us/microsoft-365/powerpoint).

Table S1. The regional proportion in terms of forest and sustainable indicators

Percentages of regions in the country (%)

		Land Area	Forest Area	CO_2 Removal	CO_2 value	NC	IW	GDP
Geographical Regions	Hokkaido	21.0	22.1	15.1	13.8	24.8	4.0	3.5
	Tohoku	18.0	18.9	18.7	21.2	18.5	6.8	6.1
	Kanto	8.7	5.6	5.8	5.4	6.9	35.4	38.8
	Chubu	17.9	18.3	17.6	17.3	17.2	17.3	17.8
	Kansai	8.9	9.0	10.4	10.3	8.1	17.9	16.7
	Chugoku	8.6	9.5	12.2	11.8	8.1	5.6	5.4
	Shikoku	5.0	5.7	6.8	7.0	4.9	2.8	2.6
	Kyushu	11.9	10.9	13.5	13.2	11.4	10.1	9.2
Cite Types	Megacities	9.0	6.6	7.4	7.3	6.5	50.0	53.1
	Regional Cores	9.7	9.3	8.9	7.6	9.9	8.3	7.9
	Local Cities	81.3	84.1	83.8	85.1	83.5	41.7	39.0

Table S2. Parameters for the height-volume relationship for Sugi and Hinoki

Region	a_1	b_1	a_2	b_2	c_1	c_2	Source
**1) Sugi（P-CJ)**							
Hokkaido,Tohoku	0.057	-1.389	6475.4	-2.902	5.339	-1.513	Matsuo (1984)
Kanto	0.072	-1.374	5062.0	-2.870	5.371	-1.496	Matsuo (1984)
Chubu	0.062	-1.352	4725.2	-2.824	5.324	-1.472	FDG (2016) (b)
Kansai, Chugoku, Shikoku	0.074	-1.388	5065.0	-2.900	5.382	-1.512	FDE (1984)
Kyushu	0.050	-1.326	773.5	-2.275	4.578	-0.949	FDF (2016)
**2) Hinoki (P-CO)**							
Hokkaido, Tohoku, Kanto, Chubu	0.069	-1.242	4672.6	-3.853	5.564	-2.046	FDG (2016) (a)
Kansai, Chugoku, Shikoku	0.054	-1.184	7663.1	-3.202	5.993	-2.018	FDE (2016)
Kyushu	0.049	-1.206	8676.3	-3.262	5.958	-2.056	FDF (2016)

Table S3. Parameters for the height-age relationship

Region	α	β	γ	Source
**1) Sugi (P-CJ)**				FFPRI (2011)
Hokkaido,Tohoku	38.853	1.085	0.016	FFPRI (2011)
Kanto	34.685	1.033	0.021	FFPRI (2011)
Chubu*	34.024	0.952	0.021	FDG (2016) (b)
Kansai	46.085	0.006	0.631	FDW (2017)
Chugoku, Shikoku	39.070	1.065	0.019	FFPRI (2011)
Kyushu	37.574	0.946	0.015	FDF (2014)
**2) Hinoki (P-CO)**				
Hokkaido, Tohoku, Kanto	23.300	0.990	0.021	FFPRI (2011)
Chubu	25.657	0.899	0.020	FDG (2016) (a)
Kansai	24.839	0.957	0.023	FDW (2017)
Chugoku, Shikoku	22.520	1.060	0.030	FFPRO (2011)
Kyushu	30.560	1.020	0.016	FDF (2014)
**3)Larix leptolepis (P-LL)**				
Hokkaido	25.170	1.060	0.039	FFPRI (2011)
Tohoku	27.610	1.086	0.032	FFPRI (2011)
Other Regions	25.810	1.124	0.037	FFPRI (2011)
**4)Abies forests (P-AP)**				
Japan	34.420	1.168	0.022	FFPRI (2011)

* Only the relationship for Sugi (P-CJ) in Chubu follows the format of $H = \alpha \cdot (1 - \exp(-\beta \cdot t))^{\gamma}$. Others all follow the format of $H = \alpha \cdot (1 - \beta \cdot \exp(-\gamma \cdot t))$.

Table S4. Parameters for the height-volume relationship for pine forests

Parameters	Value
p_1	-4.095
p_2	1.681
p_3	1.121
m	8.026
n	0.04
Source	Nakajima et al. (2010)

Table S5. The results of sensitivity analysis

Model Variant	National CO_2 Removal (Mt-CO_2/yr)
Increase the parameter α by 1%	+2.094 (+1.19%)
Change pre-afforestation land use to all grasslands	-7.991 (-4.56%)
Change pre-afforestation land use to without grasslands	+0.362 (+0.21%)

References:

Forestry Division of Ehime (FDE). *Yield tables for Sugi and Hinoki plantations in Ehime Prefecture*. https://www.jma.or.jp/jmacc/data/604_r001_pj4-2.pdf (1984) (in Japanese)

Forestry Division of Fukuoka (FDF). *Yield tables and management diagrams for forests in Fukuoka.* https://www.pref.fukuoka.lg.jp/uploaded/life/107021_17829857_misc.pdf (2014) (in Japanese)

Forestry Division of Gifu (FDG). *Yield tables and stand density management diagrams for Hinoki plantations in Gifu Prefecture* https://www.pref.gifu.lg.jp/uploaded/attachment/48142.pdf (2016) (in Japanese) (a)

Forestry Division of Gifu (FDG). *Yield tables and stand density management diagram for Sugi plantations in Gifu Prefecture* https://www.pref.gifu.lg.jp/uploaded/attachment/48141.pdf (2016) (in Japanese) (b)

Forestry Division of Wakayama (FDW). *Yield tables for Sugi and Hinoki in Wakayama Prefecture* https://www.pref.wakayama.lg.jp/prefg/070600/rinbunzaisekihyou/index_d/fil/01_zaisekihyo.pdf (2017). (in Japanese)

Forestry and Forest Products Research Institute (FFPRI). Manaul for Local Yield Table Constructing System LYCS 3.3 https://www2.ffpri.go.jp/labs/LYCS/files/index.html *(2011) (in Japanese)*

Matsuo, K. Prediction of real forest growth using regional forest stand density control diagrams Ⅰ. https://www.tokyo-aff.or.jp/uploaded/attachment/9645.pdf (1984) (in Japanese)

Nakajima, T., Matsumoto, M., Sasakawa, H., Ishibashi, S. & Tatsuhara, S. Estimation of growth parameters using the local yield table construction system for planted forests throughout Japan. *Journal of Forest Planning*. **15**, 99-108 (2010).

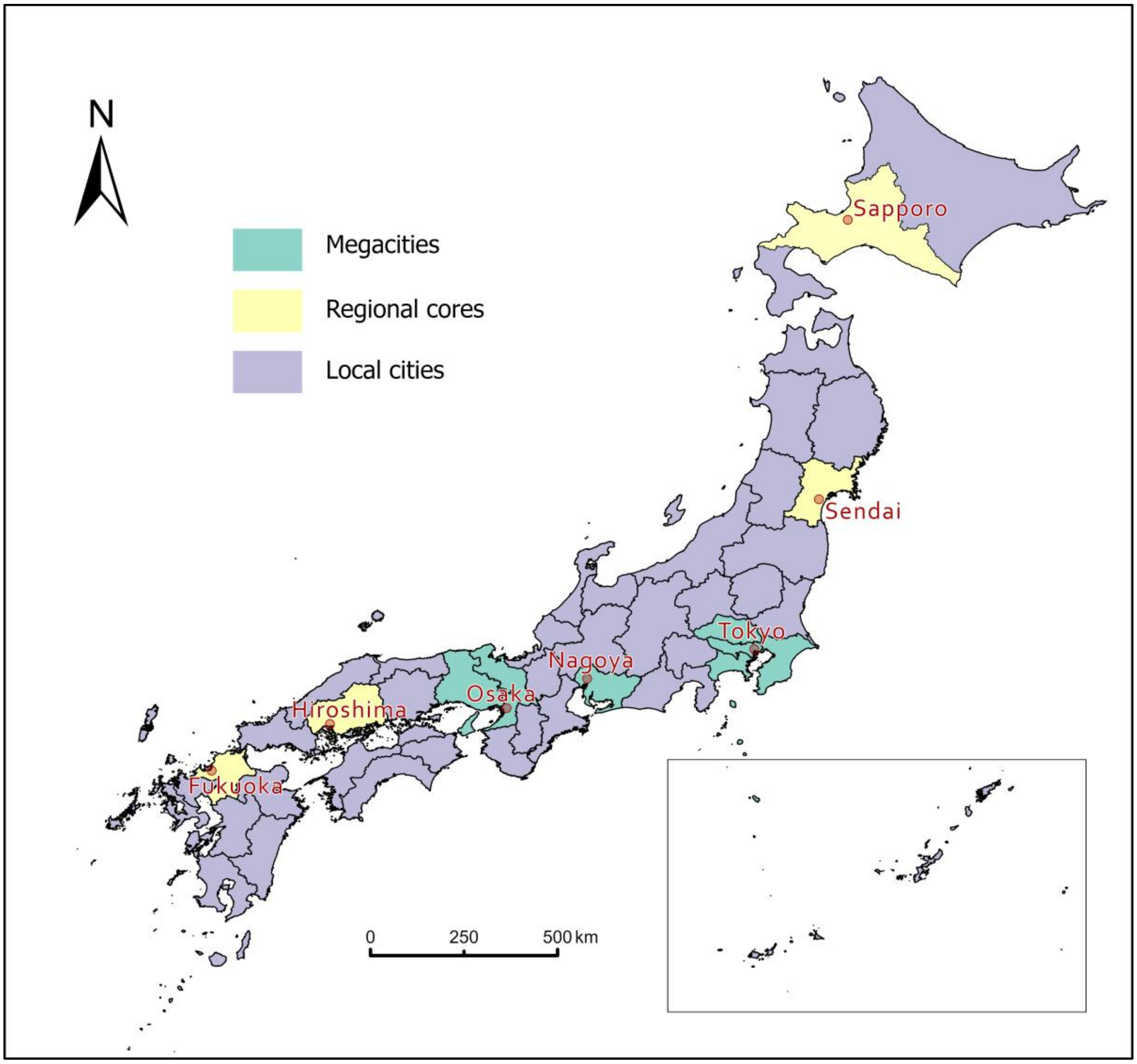

Figure S1. Megacities, regional cores, and local cities

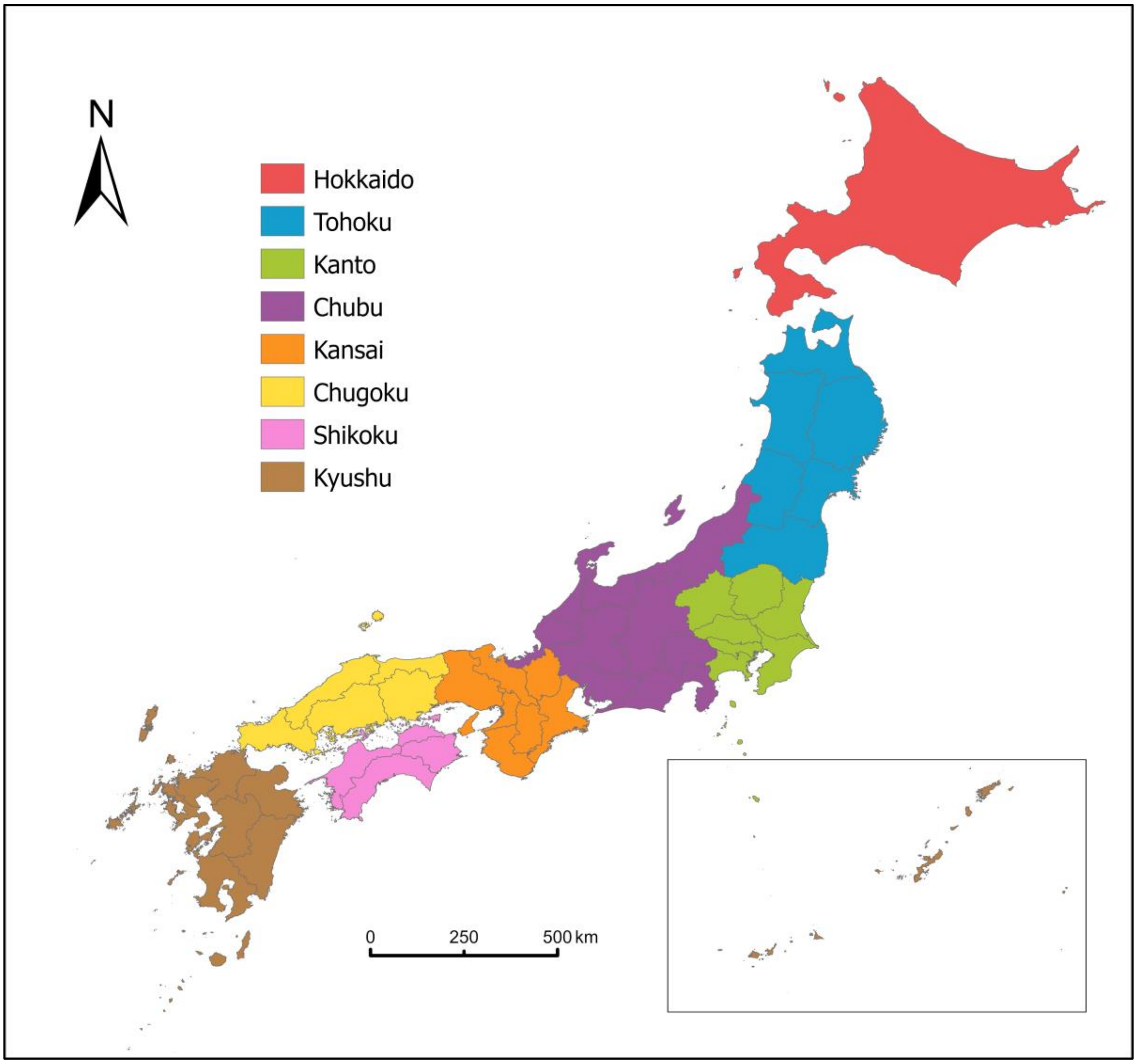

Figure S2. The geographic regions of Japan

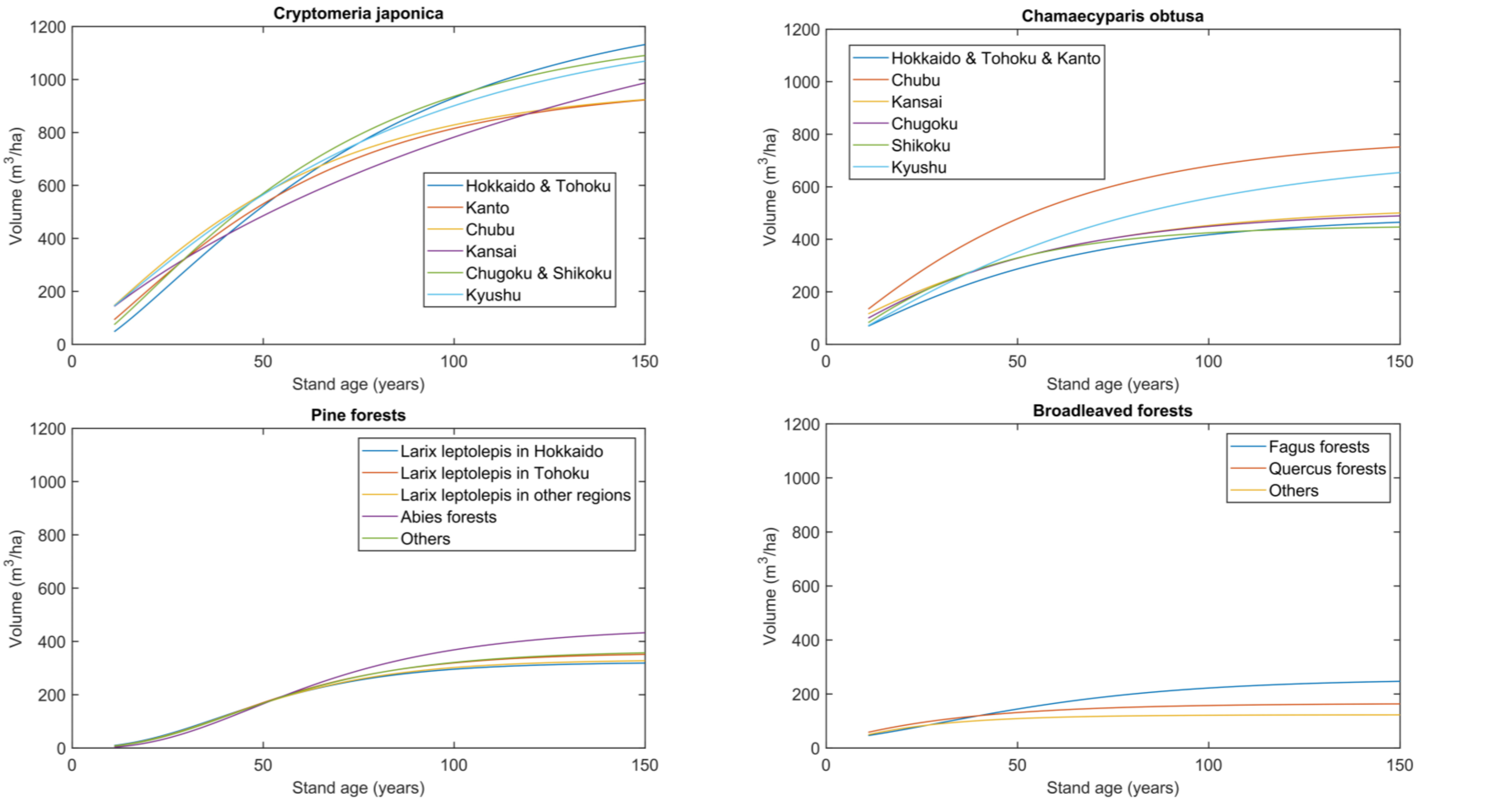

Figure S3. The curve of mean volume over stand age for different tree species and regions

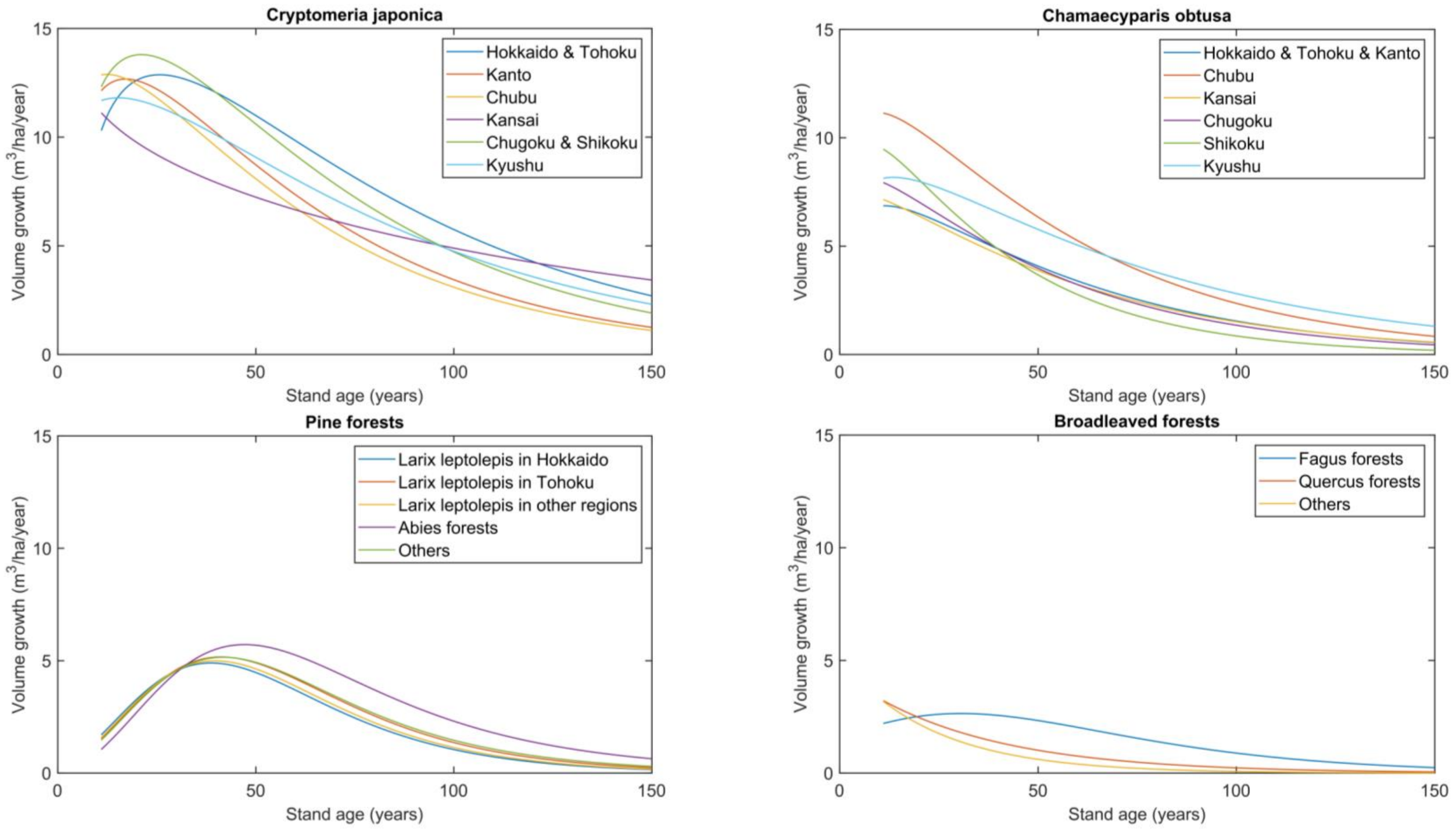

Figure S4. The curve of mean volume growth over stand age for different trees and regions

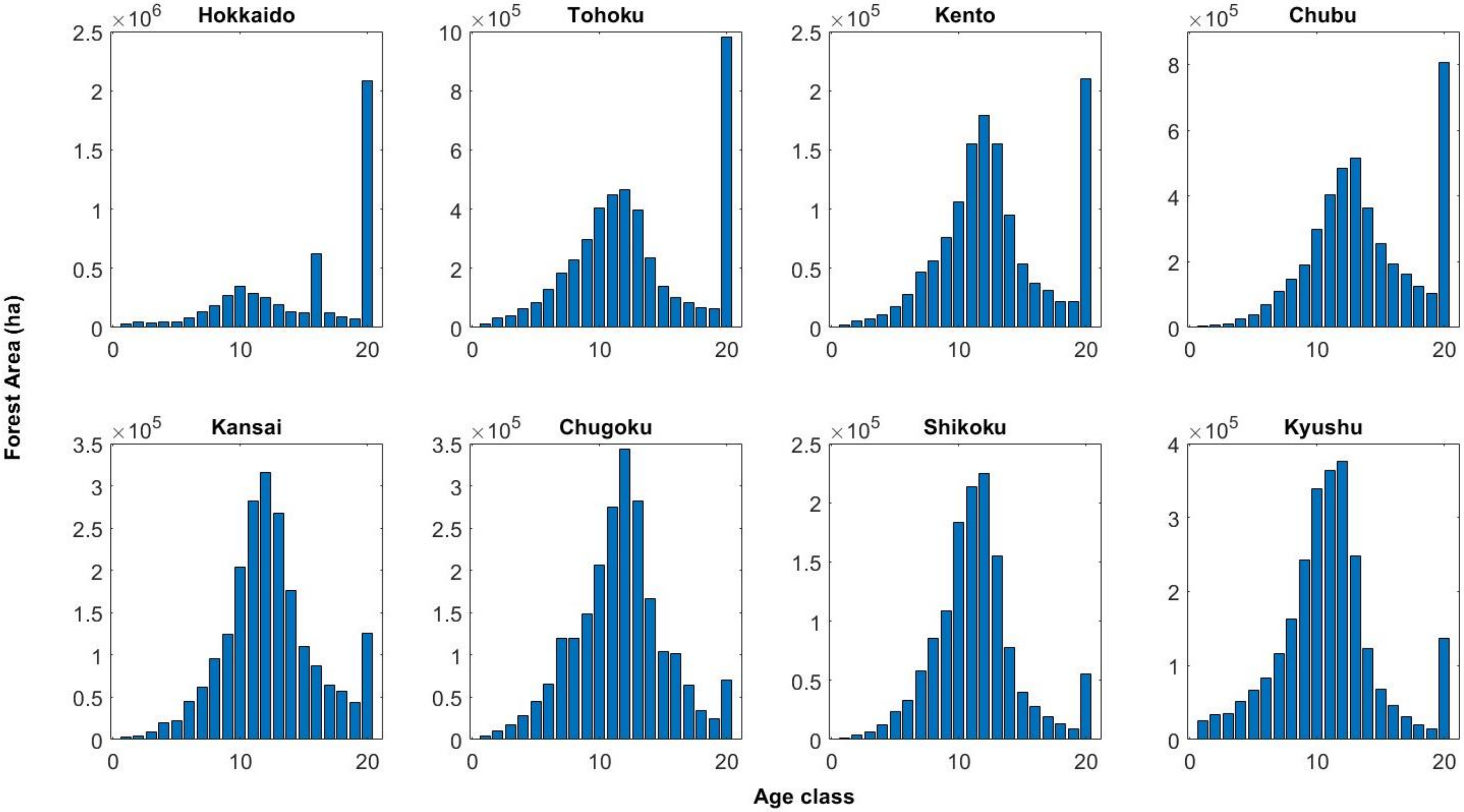

Figure S5. Tree age distribution in different regions

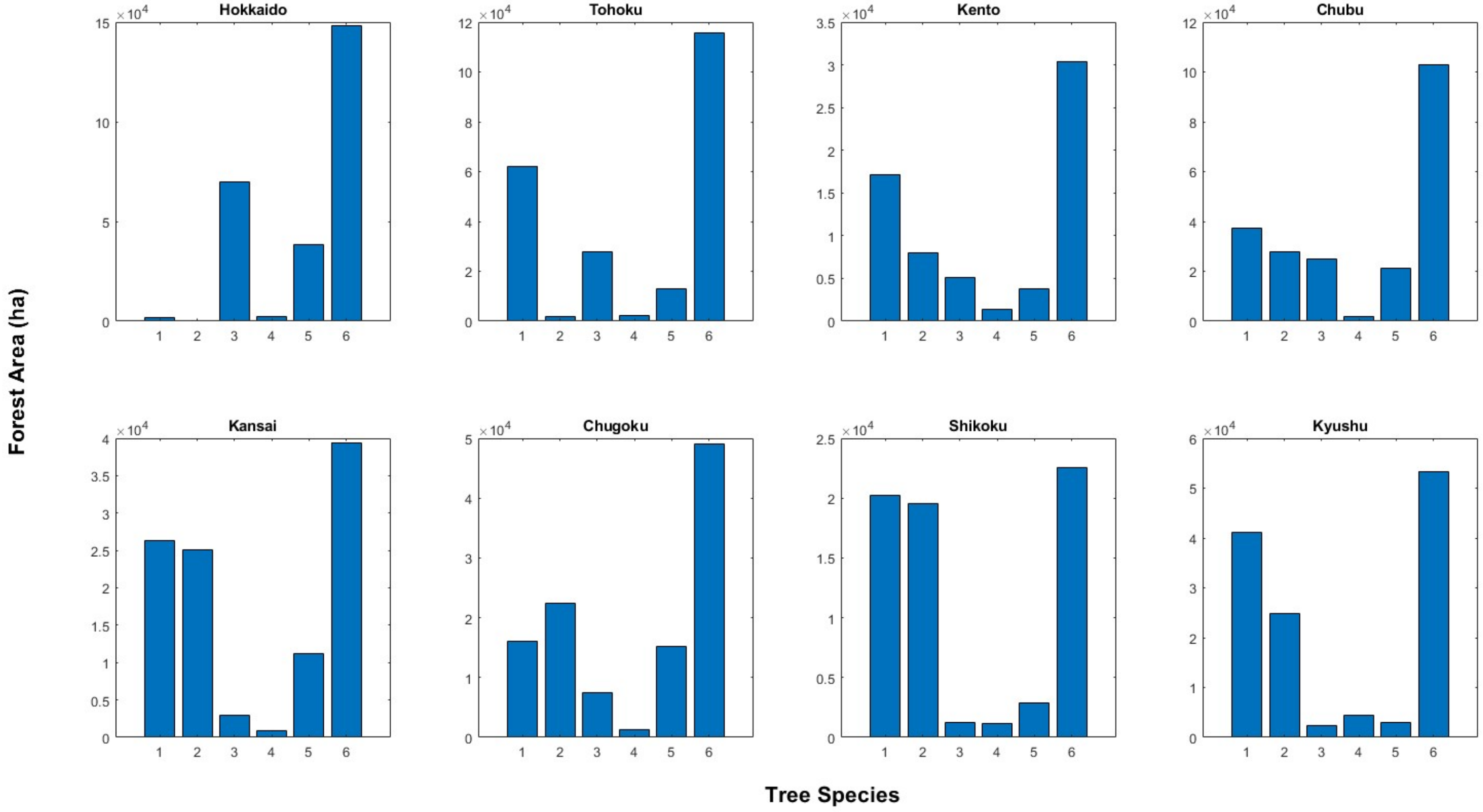

Figure S6. Tree species distribution in different regions.

The X-axis label 1 refers to P-CJ, 2 refers to P-CO, 3 refers to other needleleaf plantations, 4 refers to broadleaf plantations, 5 refers to N-NF, 6 refers to N-BF.

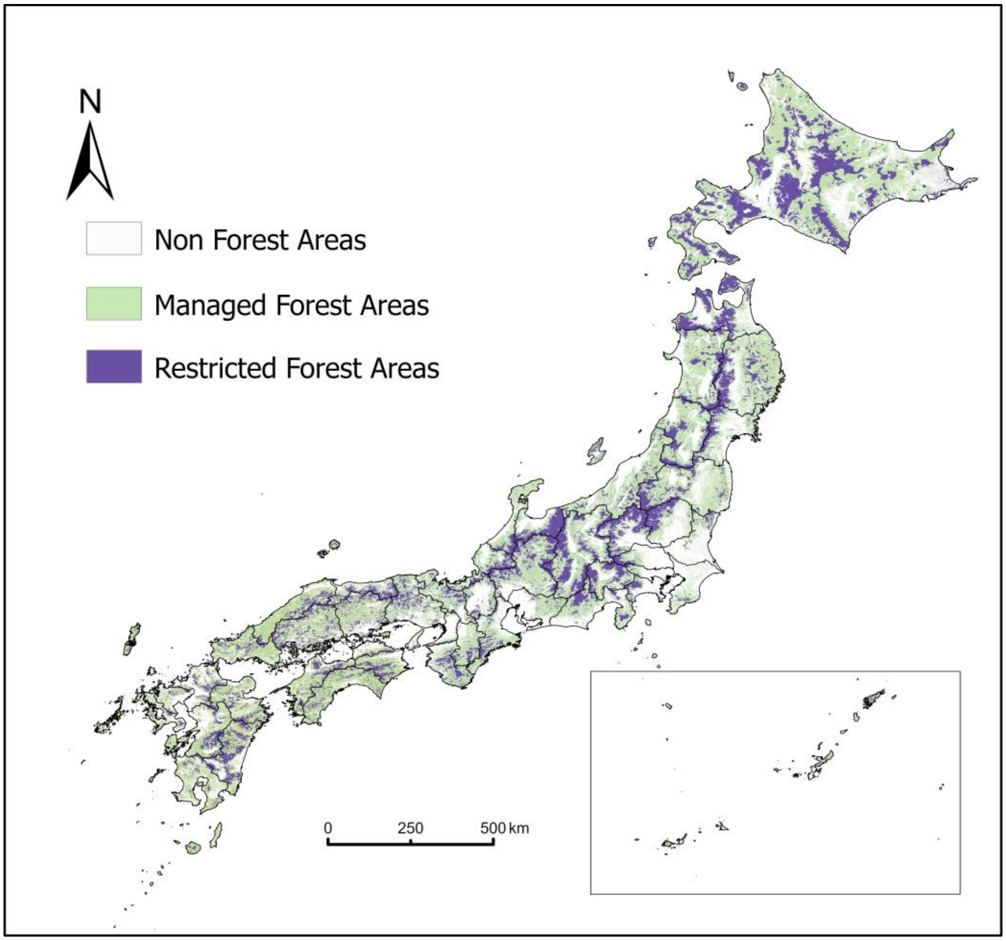

Figure S7. Forest types of Japan

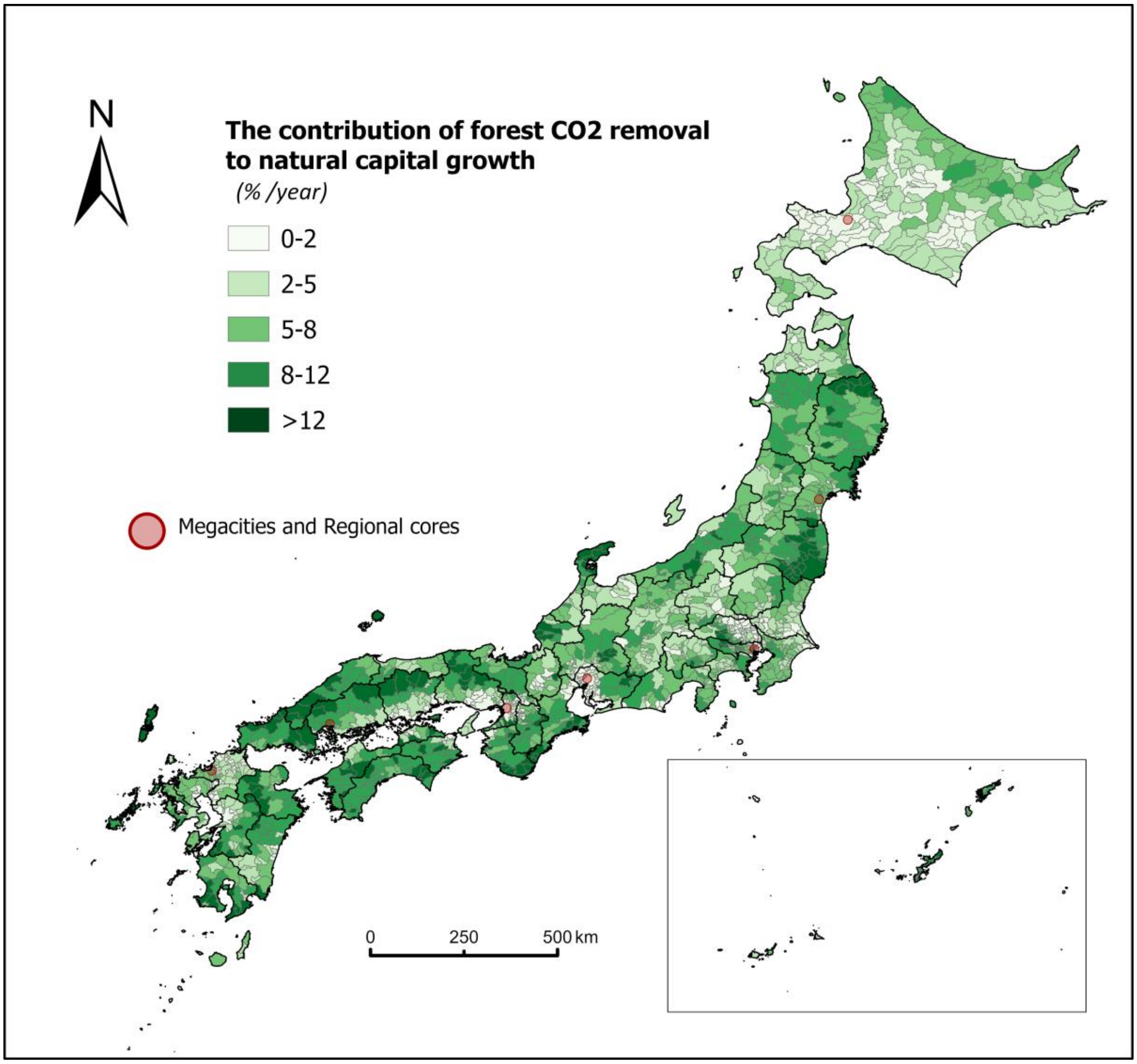

Figure S8. The contribution of forest CO_2 removal to natural capital

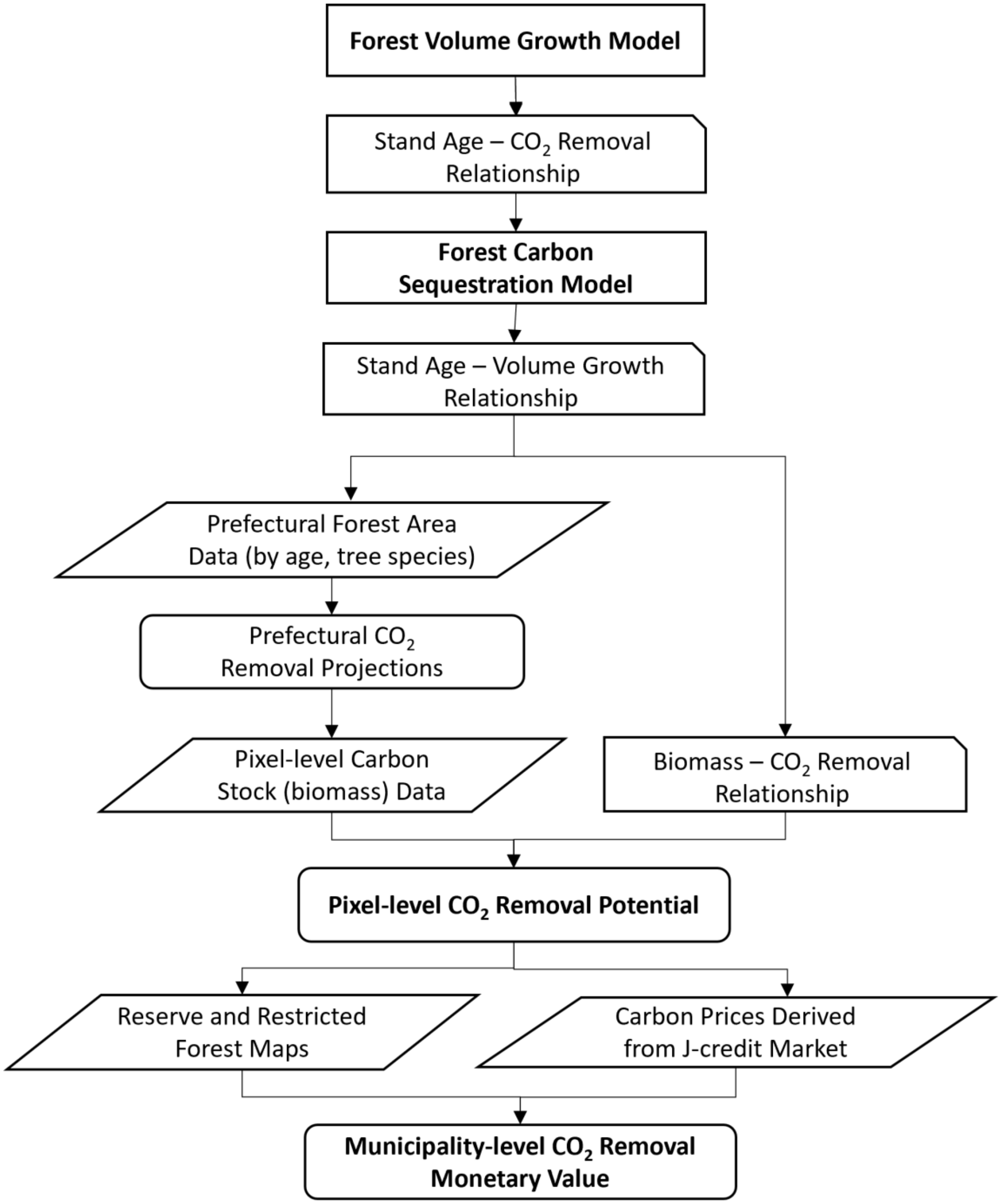

Figure S9. The flowchart for the methods

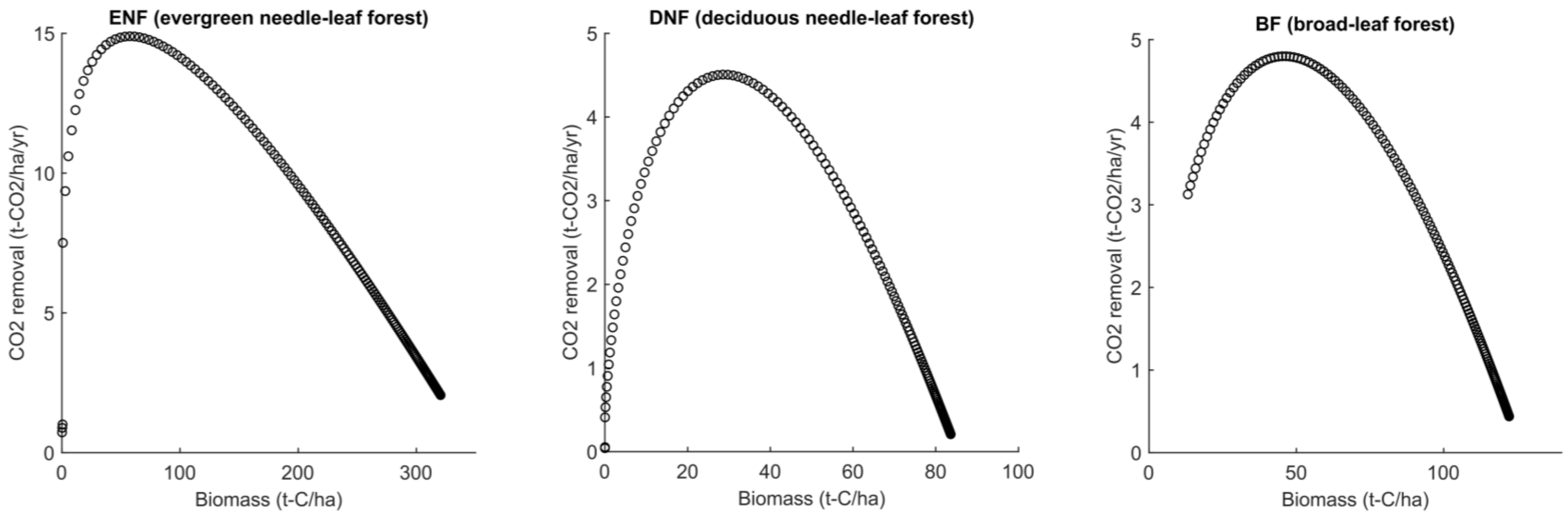

Figure S10. The relationship between CO2 removal and biomass in typical forests

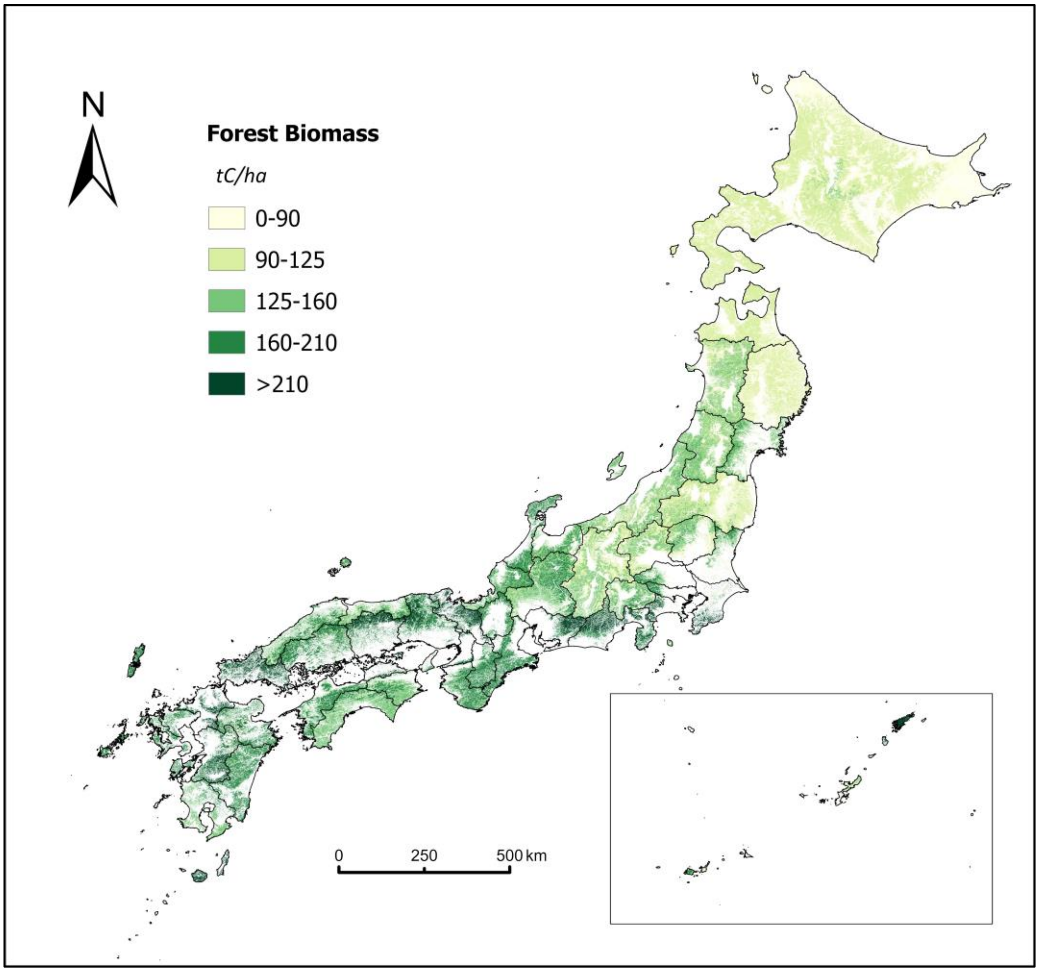

Figure S11. The forest biomass density of Japan
